# Effectiveness of Responsivity Intervention Strategies on Prelinguistic and Language Outcomes for Children with Autism Spectrum Disorder: A Systematic Review and Meta-Analysis of Group and Single Case Studies

**DOI:** 10.1007/s10803-021-05331-y

**Published:** 2021-11-15

**Authors:** Jena McDaniel, Nancy C. Brady, Steven F. Warren

**Affiliations:** 1 Life Span Institute, University of Kansas, 3001 Dole Human Development Center, 1000 Sunnyside Ave, Lawrence, KS 66045-7555 USA; 2 Department of Speech-Language-Hearing: Sciences & Disorders, University of Kansas, Lawrence, KS USA

**Keywords:** Autism spectrum disorder, Language, Meta-analysis, Prelinguistic, Responsivity

## Abstract

We conducted a systematic review to identify randomized controlled trials (RCTs) and single case research design (SCRD) studies of children with autism spectrum disorder that evaluate the effectiveness of responsivity intervention techniques for improving prelinguistic and/or language outcomes. Mean effect sizes were moderate and large for RCTs (33 studies; *g* = 0.36, 95% CI [0.21, 0.51]) and SCRD (34 studies; between-case standardized mean difference = 1.20, 95% CI [0.87, 1.54]) studies, respectively. Visual analysis (37 studies) revealed strong evidence of a functional relation for 45% of the opportunities and no evidence for 53%. Analyses of moderator effects and study quality are presented. Findings provide support for responsivity intervention strategies with more robust support for context-bound outcomes than more generalized outcomes.

**Supplementary Information:**

The online version contains supplementary material available at 10.1007/s10803-021-05331-y.

Children with autism spectrum disorder (ASD) exhibit difficulty learning language with wide variation in the nature and degree of these difficulties (Kjelgaard & Tager-Flusberg, 2001; Lord et al., 2004; Tager-Flusberg et al., 2005; Thurm et al., 2007). Concerns with communication are often one of the first developmental concerns that caregivers of children later diagnosed with ASD express (De Giacomo, & Fombonne, 1998; Howlin & Moore, 1997; Kozlowski et al., 2011). Such concerns are consistent with the observed areas of need in prelinguistic skills of children with ASD (e.g., joint attention, canonical babbling; Mundy et al., 1986; Patten et al., 2014; Sigman & Ruskin, 1999). Approximately 30% of children with ASD present with minimal verbal skills, using only a few words, even after years of intervention (Anderson et al., 2007; Tager-Flusberg & Kasari, 2013). Other individuals with ASD achieve fluent speech with large vocabularies and complete sentences (Kjelgaard & Tager-Flusberg, 2001; Tager-Flusberg & Joseph, 2003). Pragmatic language, which includes the social aspects of language, has been identified as a particular area of need for children with ASD (Lord & Paul, 1997; Wilkinson, 1998). These language difficulties may have long-term negative consequences on social and vocational outcomes, including decreased likelihood of living independently and low employment status (Billstedt et al., 2005; Howlin, 2000). Thus, determining how to best mitigate such difficulties is critical for improving long-term outcomes of individuals with ASD.

Interventions for children with ASD vary across multiple facets including theoretical basis, type of interventionist (e.g., clinicians, caregivers, peers, or their combination), degree to which interventions are child-led versus adult-led (i.e., directedness), and how the communication partner responds to communicative attempts. Investigations of the effectiveness of communication and language interventions have yielded widely varying results (e.g., Hampton & Kaiser, 2016; Reichow et al., 2018; Sandbank et al., 2020b). Because intervention studies vary in many factors (e.g., participants characteristics, outcome measures, and intervention features), systematic synthesis across studies is needed to draw conclusions. One synthesis approach is to evaluate interventions with specific components to identify active ingredients of effective interventions. This approach may enable interventionists to focus on the essential strategies. Focusing on essential strategies is especially important when caregivers serve as the interventionist because teaching too many strategies or tasks may risk overwhelming caregivers and reducing the training’s effectiveness. This systematic review and meta-analysis synthesizes and evaluates studies of interventions that use responsivity intervention strategies to target prelinguistic and language skills in children with ASD.

## Responsivity Intervention Strategies

We define responsivity intervention strategies as strategies designed to support the development of turn-taking conversations through setting up the environment to increase communication by following the child’s lead, using natural reinforcement for communicative attempts, and providing targeted input. The adult adapts their responses to the child’s focus of attention and/or on-going actions. Responsive strategies include, but are not limited to, linguistic mapping, follow-in comments, recasting, and imitating the child. Linguistic mapping occurs when an adult describes the child’s action and/or underlying message or intention (Yoder & Warren, 2002). For example, the adult says, “That’s a book,” when the child points to a book and says, “Uh”. Follow-in comments describe the child’s current focus of attention (McDuffie & Yoder, 2010). For example, the adult says, “That car is going fast,” when the child is playing with a toy car. When an adult recasts what a child says, they add grammatical or phonemic information to the child’s utterance. For example, the adult says, “That dog is big!” when the child comments, “Dog big.” When imitating a child, the adult may imitate the child’s words, sounds, gestures, and/or actions on objects. These responsivity intervention strategies can, and often do, target prelinguistic skills (e.g., joint attention and vocalizations) that are foundational to language use and conversational turn-taking.

Responsivity intervention strategies may be used independently, but they are often used within an intervention package, such as a naturalistic developmental behavioral intervention (NDBI). NDBIs combine developmental principles and applied behavior analysis (ABA) principles, follow the child’s lead, and include multiple intervention strategies to support learning and engagement (Schreibman et al., 2015). Examples include the Early Start Denver Model (ESDM), Joint Attention Symbolic Play Engagement and Regulation (JASPER), Pivotal Response Treatment (PRT), reciprocal imitation training (RIT), and Responsivity Education / Prelinguistic Milieu Teaching (RE/PMT). Responsivity intervention strategies contrast adult-driven interventions that emphasize discrete training of specific behaviors using structured prompting procedures (e.g., discrete trial training).

Responsivity intervention strategies align with multiple theories that emphasize the bidirectional interactions between children and adults in facilitating vocal and language development, including the social feedback theory (Goldstein & Schwade, 2008; Goldstein et al., 2003), social feedback loop theory (Warlaumont et al., 2014), and transactional theory of spoken language development (Camarata & Yoder, 2002; McLean & Snyder-McLean, 1978; Sameroff & Chandler, 1975). Although the details of these theories vary modestly, they all support the use of contingent caregiver responses to children’s communicative attempts to facilitate continued growth in communication and language. Thus, these theories provide support for use of responsivity intervention strategies during language intervention for children with ASD.

The social feedback theory asserts that children produce more complex and more adult-like vocalizations when adults respond contingently to them within social interactions (e.g., smiling at, moving closer to, and/or touching the infant when they vocalize) than when they respond noncontingently (Goldstein et al., 2003). The contingent nature of the response is emphasized rather than a more general response style or the quantity of input. Intervention procedures that support adults consistently responding to child vocalizations, but not responding when the child is not producing vocalizations, would align with the social feedback theory.

The social feedback loop theory emphasizes that adults are more likely to respond to children’s speechlike utterances than non-speechlike utterances and children are more likely to produce speechlike utterances when their communication partner responds to their immediately preceding utterance (Warlaumont et al., 2014). The social feedback loop theory aligns with intervention approaches that increase adults’ responses to children’s utterances as well as increasing the number of child vocalizations.

The transactional theory of spoken language development posits that caregivers provide increasingly complex input to the child as the child produces more complex communication and language acts. The relatively more complex input scaffolds continued child growth that evokes even more complex input (Camarata & Yoder, 2002). Thus, this theory supports intervention strategies that encourage adults to provide input that is contingent on and somewhat more complex than the child’s utterances.

## Relevant Prior Reviews

No known prior reviews specifically address the effects of responsivity intervention strategies on prelinguistic and language skills of children with ASD using randomized controlled trials (RCTs) and single case research design (SCRD) studies, which can address this causal question. One known systematic review and meta-analysis evaluated the effectiveness of intervention studies that addressed parent verbal responsiveness and child communication for children with or at risk for ASD (Edmunds et al., 2019). Because the meta-analysis only included five RCTs for the intervention studies, the results must be interpreted with caution. The findings identified improvement in parent verbal responsiveness but not child communication. Some of the included studies reported benefits for child communication but others did not. The limited number of studies available precluded more detailed analysis to explain the variation in results. Other reviews that have also focused on specific types of intervention (e.g., early intensive behavioral interventions [Reichow et al., 2018], parent-mediated early interventions [Oono et al., 2013], ESDM [Ryberg, 2015]) have been limited by the number and quality of relevant studies to include. These example meta-analyses included at most eight studies with at most two being RCTs.

Taking a different approach, a few other prior reviews have examined effects of broad language intervention for young children with ASD, regardless of the intervention type. These review studies were restricted to group design studies and have often included quasi-experimental studies in addition to randomized controlled trials (Hampton & Kaiser, 2016; Sandbank et al., 2020a, 2020b). Sandbank and colleagues reported a positive, but small, statistically significant mean effect size for the effects of nonpharmacological early intervention on multiple areas of development, including language, for group design studies (Sandbank et al., 2020a, 2020b). Similarly, Hampton and Kaiser (2016) reported a small, significant mean overall effect size (*g* = 0.26, 95% CI [0.11, 0.42]) for spoken language outcomes. Some reviews included children at risk for ASD, rather than only children diagnosed with ASD (Edmunds et al., 2019).

## Factors that May Influence the Presence and Strength of Intervention Effects

Some of the reviews described above have investigated several factors that may influence the presence and strength of intervention effects. The results have often been mixed, which supports the need for continued investigation to reach a consensus. These variables include the interventionist, time in intervention, proximity of outcome measures, boundedness of outcome measures, risk of correlated measurement error, and publication bias.

## Interventionist

ASD interventions may be implemented by a variety of individuals including caregivers, clinicians, and/or peers. Some interventions are implemented by multiple individuals, such as a caregiver and a clinician simultaneously with varying levels of caregiver training provided (e.g., Gengoux et al., 2019; Roberts et al., 2011; Vivanti et al., 2014). Logically, a child may benefit from both the caregiver spending relatively more time with the child during the day to implement therapeutic strategies and the clinician’s expertise implementing and adapting strategies. Both Sandbank et al. (2020b) and Hampton and Kaiser (2016) reported stronger effects for intervention implemented by caregivers and clinicians than those implemented by caregivers alone. Sandbank et al. (2020b) also identified a larger effect size for interventions implemented by clinicians alone than those by caregivers alone, but Hampton and Kaiser (2016) did not find similar differences. Fuller and Kaiser (2020) did not identify a differential effect by interventionist. These three meta-analyses included responsive language interventions, but not exclusively.

## Time in Intervention

School-based speech-language pathologists report providing more intensive intervention services for children with severe communication needs (Brandel & Frome Loeb, 2011). Yet, there is relatively little relevant data regarding whether more intensive intervention yields greater language gains for children with ASD, despite its intuitive appeal (Baker, 2012; Warren et al., 2007). A number of meta-analyses have failed to identify total intervention dosage as a moderator of effect size for speech-language outcomes in the meta-analysis for children with ASD (Fuller & Kaiser, 2020; Hampton & Kaiser, 2016; Sandbank et al., 2020b). The current synthesis provides an opportunity to test whether a greater amount of time in intervention improves prelinguistic and language outcomes for interventions that use responsivity intervention strategies. As described by Warren et al. (2007), intervention intensity can be quantified in multiple ways. Because we anticipated limited reporting of the necessary details to calculate cumulative intervention intensity, we selected time in intervention (minutes per week times number of weeks of intervention) as the intensity variable.

### Proximity of Outcome Measure

Proximal outcome measures assess skills taught directly during the intervention. Distal outcome measures assess skills beyond what was taught directly. As predicted, Yoder et al. (2013) found significantly greater probability of an effect on social communication for proximal outcome measures (63%) than distal outcome measures (39%) for children with ASD.

### Boundedness of Outcome Measure

Boundedness of outcome measures refers to the degree to which the occurrence of the outcome behavior depends on the intervention context (e.g., same setting, materials, and/or communication partner; Yoder et al., 2013). Context-bound outcome measures are measured in situations very similar to the treatment sessions (e.g., evaluating the number of intentional communication acts during treatment sessions with the interventionist). In contrast, generalized characteristics are measured in situations that vary from the treatment context in setting, materials, and/or communication partner (e.g., number of intentional communication acts with an unfamiliar clinician during a session in which the intervention strategies are not used). Potentially context-bound outcome measures may show changes that are possibly limited to the treatment context (e.g., standardized caregiver report measure for a caregiver-implemented intervention). Yoder et al. (2013) found greater probability of a significant effect on social communication for context-bound outcome measures (82%) than generalized characteristics (33%). Boundedness also moderated the mean effect size for the effectiveness of early intervention on social communication skills of children with ASD (Fuller & Kaiser, 2020).

### Risk for Correlated Measurement Error

Correlated measurement error (CME) systematically elevates the true score for the predicted superior group or phase over the control group or phase (Yoder et al., 2018). Intervention studies are at risk for CME (a) when the outcome measure coder is not blind to treatment assignment and (b) when interventionists (including caregivers) provide the intervention and serve as the examiner when the outcome measure is assessed.

### Publication Bias

Publication bias occurs “when published research on a topic is systematically unrepresentative of the population of completed studies on that topic” (Rothstein, 2008, p. 61). We test for this known risk for meta-analyses by comparing effect sizes of published versus unpublished studies. This examination is a feature of well-designed meta-analyses.

## The Current Literature Synthesis

The purpose of this systematic review and meta-analysis is to describe the current state of the literature for responsivity intervention strategies aimed at improving prelinguistic and language skills of children with ASD with an eventual outcome of shaping the direction of future research studies and clinical practice. Most of the prior reviews are systematic, but do not employ meta-analytic techniques (Mancil et al., 2009; McConachie & Diggle, 2007; Verschuur et al., 2014). Our review uses meta-analytic techniques to determine the mean effect size not only for group design studies, specifically RCTs, but also for SCRD studies. Including SCRD studies is important because many studies of responsive interventions have used single case designs. SCRD studies avoid the need for large samples required for RCTs to make causal conclusions by each participant serving as their own control and by using specific designs to control for threats to internal validity (Ledford & Gast, 2018). We restricted the research synthesis to RCTs and SCRD studies because those designs permit causal conclusions, unlike quasi-experimental or other non-randomized group designs. This design requirement combined with the quality analysis enabled this research synthesis to focus on studies with relatively higher quality of evidence. We conducted two separate analyses—one for RCTs and a second for SCRD studies. We then descriptively discuss the results of the two analyses. The review is registered with PROSPERO (CRD42020157374).

## Research Questions

To provide a comprehensive review of the literature, we included RCTs and SCRD studies that met quality criterion. We addressed two primary research questions, separately for the RCT and SCRD studies: (1) Is the mean effect size for interventions that use responsivity intervention strategies on communication and/language skills in children with ASD greater than zero? (2) Does the mean effect size vary by interventionist, time in intervention, proximity or boundedness of the outcome measure, risk for CME, or publication bias? We also assessed study quality descriptively using the Revised Cochrane risk-of-bias tool for randomized trials (RoB 2; Higgins et al., 2019) and What Works Clearinghouse standards for SCRDs (What Works Clearinghouse, 2016). Both tools address potential bias from multiple sources including, but not limited to, the study design, completeness of the data, and data analysis.

## Methods

### Search Strategy

Our comprehensive search strategy included multiple search methods. The main search utilized electronic databases. We searched PubMed on October 18, 2019 and the Education Database, ERIC, Health & Medical Collection, Linguistics and Language Behavior Abstracts, Linguistics Database, ProQuest Dissertations & Theses Global, Psychology Database, PsycINFO, and Social Science Database in ProQuest and the Cumulative Index of Nursing and Allied Health Literature (CINAHL) on October 19, 2019. See Supplementary Information 1 for an example search.

For supplementary searches, the first author hand searched table of contents for the past year for journals that contributed at least five articles to the full text screening from the main database search (i.e., Autism, Journal of Autism and Developmental Disorders, Journal of Child Psychology and Psychiatry). The first author also screened abstracts from the two prior conferences for the Gatlinburg Conference on Intellectual and Developmental Disabilities, International Meeting for Autism Research, and Society for Research in Child Development to identify findings that may not yet be in publication. Finally, the first author scanned reference lists and conducted forward searches for included studies. The supplementary searches were completed on March 28, 2020.

The primary coder (first author) screened 100% of the identified reports. Trained research assistants independently screened 25% of the reports at the title and abstract level and the full text level. The primary coder (first author) was blind to which reports would be coded for reliability. To prevent coder drift, discrepancy discussions were completed regularly. Point-by-point agreement for inclusion or exclusion (i.e., agreements divided by total number of reports) was 89% at the title and abstract level and 87% at the full text level. We used the primary coder’s decisions for inclusion.

### Inclusion Criteria

#### Population

Study participants had to be children diagnosed with ASD with a mean or median age under 18 years, 0 months at intervention initiation. We included numerous diagnostic search terms due to the change in diagnostic criteria and terminology in recent decades. Participants with autism spectrum disorder(s), autism, autistic disorder, pervasive developmental disorder–not otherwise specified, high-functioning autism, and Asperger’s disorder/syndrome were included if they met other inclusion criteria. We only included children at “high-risk” for ASD (e.g., infant siblings of children with ASD) if they were later diagnosed with ASD. For RCTs, each group was required to contain at least five participants to permit calculation of an effect size.

#### Intervention

We included studies that tested the effects of a behavioral intervention that used responsivity intervention strategies designed to improve prelinguistic and/or language skills in children with ASD. The interventionist responds to the child’s communicative attempts and provides targeted prelinguistic and/or language input. Responsivity intervention strategies include but are not limited to an adult or peer imitating the child’s vocalizations or spoken words, recasting the child’s verbal or nonverbal communication act, contingent responses to child vocalizations that continues the turn-taking exchange, and follow-in comments. We did not exclude studies based on the type of interventionist (e.g., caregivers, clinicians, teachers, and/or peers).

#### Comparison

For RCTs, the treatment group (the group that received responsivity intervention strategies) must be compared with a randomly assigned control group that does not receive responsivity intervention strategies. The control group may vary in type including, but not limited to, other intervention strategies that do not use responsivity intervention strategies, a business-as-usual condition, or a waitlist control. For the SCRD studies, a baseline or alternative intervention condition serves as the comparison, depending on the study design.

#### Outcomes

Studies must report at least one prelinguistic skill and/or language measure for the child participants with ASD. Outcome measures may be expressive language (e.g., expressive vocabulary, mean length of utterance, and requests), receptive language (e.g., receptive vocabulary and following directions), or prelinguistic skills (e.g., directed vocalizations, joint attention, and gestures).

For the RCTs, each report must include at least one group mean difference effect size or sufficient data to calculate one for an eligible outcome measure. For applicable SCRD studies, we calculated the between-case standardized mean difference (BC-SMD) because it applies to multiple baseline across participants studies (the most common design of this review), quantifies magnitude and consistency of change, and is more similar to group design effect sizes than within-case effect sizes (Hedges et al., 2012, 2013; Pustejovsky et al., 2014; Valentine et al., 2016). We present the RCT and SCRD study results separately to permit comparison of the RCT results with prior meta-analyses and to avoid differences in weighting of sample sizes across study types (Valentine et al., 2016).

### Exclusion Criteria

To maintain an appropriately narrow focus, literacy, vocal stereotypy, and challenging behavior outcomes were excluded. We also excluded outcome measures that focused on the interventionist’s performance (e.g., number of adult conversational turns, prompts to the child, or use of intervention strategies). We excluded studies not written in English due to lack of translation resources. Studies were not excluded based on the language of the participants or the publication date. At the final stage of the full text screening, we excluded SCRD studies that failed to meet quality standards from the qualitative and quantitative analyses because failing to meet those standards prevents interpretation of the findings (What Works Clearinghouse, 2016). Broadly, the following criteria must be met to demonstrate an intervention effect: (a) graphical display of the data, (b) at least three attempts to demonstrate an effect and (c) a sufficient number of data points per phase (e.g., at least three data points per phase to meet with reservations and at least five data points per phase to meet without reservations for multiple baseline, multiple probe, and ABAB [reversal/withdrawal] designs; What Works Clearinghouse, 2016). Multiple baseline and multiple probe designs must also have sufficiently overlapping baselines across tiers. Failure to meet all these criteria resulted in exclusion from the qualitative and quantitative analyses. For additional details, refer to the What Works Clearinghouse Study Review Guide Instructions for Reviewing Single-Case Designs Studies (What Works Clearinghouse, 2016).

### Study Selection

**Fig. 1:**
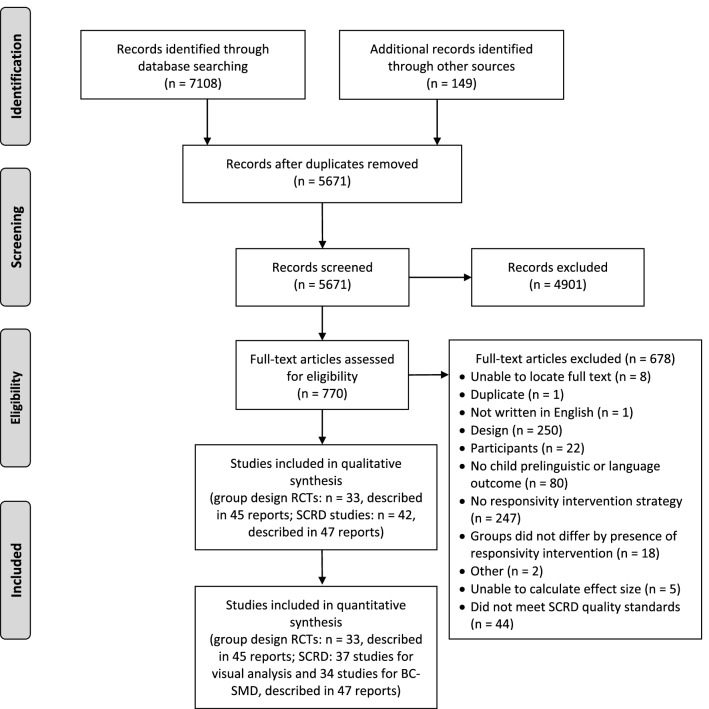
Preferred Reporting Items for Systematic Reviews and Meta-Analyses (PRISMA) flow diagram. BC-SMD = between-case standardized mean difference; RCTs = randomized controlled trials; SCRD = single case research design

As shown in the Preferred Reporting Items for Systematic Reviews and Meta-Analyses (PRISMA) flow diagram (Fig. 1), database searches yielded 7108 records and other sources yielded 149 records. After eliminating duplicates and screening the titles and abstracts, 770 records remained. During the full text screening, independent coders eliminated studies in the order listed in Fig. 1. For the SCRD studies, the final inclusion criterion was meeting quality standards with or without reservations. The search yielded 33 RCTs that were described in 45 reports and included 294 relevant effect sizes and 42 SCRD studies that were described in 47 reports. Thirty-seven SCRD studies included sufficient graphical information for visual analysis (91 relevant opportunities to detect a functional relation) and 34 permitted extractions of at least one BC-SMD effect size (69 total BC-SMD effect sizes).

### Coding the Studies

All reports were coded by the primary coder (first author) and trained research assistants using a detailed coding manual (available from first author upon request). For the RCTs, point-by-point agreement for data extraction and bias coding was 94% and 90%, respectively. For the SCRD studies, point-by-point agreement for quality coding, data extraction, and visual analysis was 80%, 92%, and 80% respectively. Discrepancies were resolved by consensus. Consensus coding was used for all analyses.

Report level features included publication status, report type, country, spoken language of the participants, and percent of participants who are monolingual. Effect size level features included sample size (for ASD group and control group for RCTs), sex, age, intervention, interventionist, time in intervention, outcome measure(s), and effect size. The total time in intervention is the number of minutes per week multiplied by the number of weeks of intervention. For caregiver-implemented interventions, the amount of time is based on structured intervention time, not all waking hours, even though a caregiver may implement at least some strategies throughout the entire day. We categorized the outcome measures as distal or proximal and context-bound, potentially context-bound, or a generalized characteristic.

For risk of bias for RCTs, we used the Revised Cochrane risk-of-bias tool for randomized trials (RoB 2; Higgins et al., 2019). We rated each study for low, moderate, or high risk of bias for randomization process, deviations from intended interventions, missing outcome data, measurement of outcome, selection of reported result, and overall. In addition, coded study quality features include risk for CME, method of handling missing data, and use of blind assessors. For quality coding for the SCRD studies we used guidelines provided by the What Works Clearinghouse Study Review Guide Instructions for Reviewing Single-Case Designs Studies (What Works Clearinghouse, 2016). Studies that did not meet quality standards with or without reservations were excluded from the meta-analysis. Remaining studies were categorized as meeting standards with versus without reservations.

### Analytic Strategies

#### Effect Size

For the RCTs, we calculated the standardized mean difference (*d*) for independent groups (i.e., mean of responsivity intervention group minus the mean of the control group divided by the within-groups standard deviation) for each relevant outcome measure (Borenstein et al., 2009). Consistent with current meta-analytic techniques, we then used a correction factor to convert to Hedges’ *g* to address the tendency for *d* to overestimate the standardized mean difference for small samples (Borenstein et al., 2009).

For the SCRD studies, we digitized the data (Huwaldt, 2010) to convert the graphical data into numerical data. We then calculated the BC-SMD using the online single-case design hierarchical linear model (scdhlm) web application (Valentine et al., 2016). For consistency, all effect sizes were calculated with restricted maximum likelihood estimation and with fixed and random effects permitted for the baseline and intervention phases (Valentine et al., 2016).

#### Visual Analysis for SCRD Studies

For visual analysis for SCRDs, we followed guidelines by Kratochwill et al. (2010), *What Works Clearinghouse Single-Case Design Technical Documentation*, for determining whether an effect is present. The visual analysis focuses on level, trend, variability, immediacy of the effect, overlap, and consistency of data patterns across similar phases.

#### Robust Variance Estimation

Because traditional meta-analytic techniques assume that all effect sizes are independent, only one effect size per sample can be used. In contrast, robust variance estimation permits inclusion of multiple effect sizes per study (Hedges et al., 2010; Tanner-Smith & Tipton, 2014). We used a random effects model with approximately inverse variance weights to address the dependency of multiple effect sizes per study via the robumeta.ado file from the Stata Statistical Software Components archive.

#### Moderator Analyses for Putative Moderators of Intervention Effects

We used meta-regression with robust variation estimation to conduct the planned moderator analyses. To evaluate variation in effectiveness across studies that use responsivity intervention strategies, we tested six moderators as shown in Table 1. After examining intercorrelations among putative moderators, all moderators were tested independently.

**Table 1:** Putative moderators of intervention effect by type

Intervention implementation	Type of outcome measure	Study quality
Interventionist	Boundedness	Publication bias
Time in intervention	Proximity	Risk for correlated measurement error

## Results

### Study Characteristics

For the RCTs, Tables 2 and 3 display participant characteristics and intervention features. At least 897 unique participants (accounting for possible overlap between studies) are included in at least one effect size. Participants’ mean age at study initiation was 43.01 months (*SD* = 17.97 months). A variety of interventions were implemented. Joint attention intervention / JASPER (8 studies) and PRT (6 studies) were most common. JASPER targets joint attention, play, and imitation through a combination of behavioral and developmental principles (Chang et al., 2016; Goods et al., 2013; Kasari et al., 2006). PRT is designed to target “pivotal” areas using ABA principles and to train caregivers in the strategies to do so (Hardan et al., 2015). Caregivers were the most common interventionist (17 studies). Table 4 displays details for effect size features, including outcome measures. The included studies used a wide variety of outcome measures and varied in the number of effect sizes per study, ranging from 1 to 78.

**Table 2:** Participant characteristics for included randomized controlled trials

Reference	Pub	Loc	*n*	T1 *M* age (mths)	T1 Developmental Level *M* (*SD*)	ASD Severity *M* (*SD*)
Independent samples						
Boyd et al., (2018)	Yes	USA	82	49	MSEL composite SS: 54.03 (11.31)	ADOS severity: 7.10 (1.91)
Carter et al., (2011)	Yes	USA	28	21	MSEL EL AE: 8.22 (6.01)	NR
Chang et al., (2016)	Yes	USA*	38	49	MSEL MA: 34.52 (10.73)	ADOS severity: 7.06 (1.26)
Clionsky, (2012) & Ginn et al., (2017)	Mixed	USA	15	57	NR	CARS-II severity: 49.67 (7.16)
Drew et al., (2002)	Yes	UK	12	21	GSMD NVIQ: 88.1 (11.2)	ADI RSI: 19.6 (3.0)
Gengoux et al., (2019)	Yes	USA	23	50	MSEL composite SS: 49.9 (1.8)	CGI-S: 5.4 (0.5)
Goods et al., (2013)	Yes	USA*	5	49	MSEL DQ: 37.70 (15.21)	NR
Hardan et al., (2015)	Yes	USA*	25	49	MSEL DQ: 52.8 (16.4)	CGI-S: 5.2 (0.9)
Kaale et al., (2012)	Yes	Norway	34	48	MSEL DQ: 53.3 (19.2)	NR
Kasari et al., (2010)	Yes	USA*	19	30	MSEL DQ: 64.80 (5.35)	NR
Kasari et al., (2014)	Yes	USA	51	42	MSEL MA: 23.6 (11.6)	ADOS severity: 7.23 (1.6)
Kasari et al., (2015)	Yes	USA*	43	31	MSEL DQ: 68.0 (20.3)	NR
Landa et al., (2011)	Yes	USA	24	29	MSEL VR T-score: 27.50 (8.27)	NR
Lawton & Kasari, (2012b)	Yes	USA	9	46	MSEL MA: 30.3 (5.01)	NR
Mohammadzaheri et al., (2014)	Yes	Iran	15	111	Summary score NR	NR
Nefdt et al., (2010)	Yes	USA*	13	39	NR	NR
Openden, (2005)	No	USA	16	58	NR	NR
Rahman et al., (2016)	Yes	India & Pakistan	29	64	VABS AB SS: 62.53 (12.15)	NR
Schertz et al., (2013)	Yes	USA	11	25	NR	NR
Schertz et al., (2018)	Yes	USA	64	25	MSEL composite SS: 104.48 (35.16)	ADOS-T severity: 16.36 (3.45)
Schreibman & Stahmer, (2014)	Yes	USA*	20	30	Summary score NR	NR
Siller et al., (2013)	Yes	USA	34	58	MSEL VR AE: 26.6 (9.4)	ADOS Social Affect: 14.7 (3.3)
Turner-Brown et al., (2019)	Yes	USA	32	30	MSEL composite SS: 62.53 (16.12)	PIA total: 2.82 (0.61)
Venker et al., (2012)	Yes	USA*	7	41^†^	MSEL VR AE: 28.79 (13.80)^†^	ADOS severity: 8 (2.13)^†^
Vernon et al., (2019)	Yes	USA*	12	38	MSEL composite SS: 76.08 (20.38)	ADOS severity: 7.00 (1.48)
Warreyn & Roeyers, (2014)	Yes	Belgium	18	69	PIQ: 79.38 (16.19)	NR
Wong, (2013)	Yes	USA*	18	NR	NR	NR
Shared samples						
Aldred et al., (2004) sample	Yes	England	14	48–51	VABS AB composite: 25.6 (9.2)	ADI median: 16.5; range 11–21
Dawson et al., (2010) sample	Mixed	USA	15–24	24	MSEL NVIQ: 83.6 (13.3)	ADOS severity: 7.2 (1.7)
Green et al., (2010) sample	Yes	UK	74–77	45	MSEL NVIQ AE: 27.0 (10.0)	ADOS severity: 8.0 (1.4)
Kasari et al., (2006) sample	Yes	USA	15–20	42–43	MSEL DQ: 58.30 (17.18)	NR
Ingersoll, (2010) sample	Yes	USA*	11–14	39–41	Bayley NV MA: 20.8 (6.6)	NR
Rogers et al., (2012) sample	Yes	USA	49–51	21	MSEL DQ: 66.89 (18.61)	ADOS severity: 7.20 (1.94)

*Location based on the first author because it was not stated explicitly

^†^Value includes control group because not reported for only treatment group

*AB*  adaptive behavior, *ADI*  Autism Diagnostic Interview (Lord et al., 1994), *ADOS*  Autism Diagnostic Observation Schedule (Lord et al., 1999), *AE * age equivalent, *ASD*  autism spectrum disorder, *Bayley*  Bayley Scales of Infant & Toddler Development (Bayley, 2005), *CARS-II*  Childhood Autism Rating Scale (Second Edition; Schopler et al., 2010), *CGI-S*  Clinical Global Impressions Scale – Severity (Guy, 1976), *DQ*  developmental quotient, *EL*  expressive language, *GSMD*  Griffiths Scale of Mental Development—D and E scales (Griffiths, 1986), *Loc* location, *M*  mean, *MA*  mental age, *MSEL*  Mullen Scales of Early Learning (Mullen, 1995), *mths*  months, *NR*  not reported, *NV*  nonverbal, *NVIQ*  nonverbal intelligence quotient, *PIA*  Parent Interview for Autism-Clinical Version (Stone et al., 2003), *PIQ*  performance intelligence quotient, *Pub*  published, *RSI*  Reciprocal Social Interaction, *SS*  standard score, *T1*  Time 1 / prior to intervention, *UK*  United Kingdom, *USA*  United States of America, *VABS*  Vineland Adaptive Behavior Scales (Sparrow et al., 1984), *VR*  Visual Reception

**Table 3:** Intervention features of included randomized controlled trials

Reference	Intervention	Ind. or Group	Amount of Intervention	Duration	Interventionist	Comp. Group
Independent samples						
Boyd et al., (2018)	ASAP	Both	In classroom; unable to determine specific amount	School year	Educator	TAU
Carter et al., (2011)	HMTW	Ind	8 group sessions for caregivers only & 3 in-home caregiver-child sessions	3.5 mths	Caregiver	TAU
Chang et al., (2016)	JASPER (classroom)	Both	In classroom; unable to determine specific amount	2 mths	Educator	DT
Clionsky, (2012) & Ginn et al., (2017)	CDIT	Ind	75-min session/wk & 5 min/day by caregivers	8 wks	Caregiver	DT
Drew et al., (2002)	Social-pragmatic JA focused parent training programme	Ind	Unable to determine specific amount	Unable to determine	Caregiver	TAU
Gengoux et al., (2019)	PRT	Ind	Wks 1–12: Weekly 60-min parent training session & 10 h clinician-delivered in-home intervention for child; Wks 12–24: Monthly 60-min parent training sessions & 5 h clinician-delivered in-home intervention for child	24 wks	Caregiver & clinician	DT
Goods et al., (2013)	JASPER	Ind	Two 30-min sessions/wk	12 wks	Clinician	TAU
Hardan et al., (2015)	PRT (group)	Ind	8 90-min parent-only group sessions & 4 60-min parent–child individual sessions with clinician		Caregiver	CE
Kaale et al., (2012)	JA intervention	Ind	2 20-min session 5 days/wk	8 wks	Educator	TAU
Kasari et al., (2010)	JE intervention	Ind	3 sessions/wk to train caregiver	8 wks	Caregiver	DT
Kasari et al., (2014)	JASPER	Ind	2 1-h in-home caregiver coaching sessions/wk	12 wks	Caregiver	CE
Kasari et al., (2015)	JASPER	Ind	2 30-min sessions/wk to train caregiver	10 wks	Caregiver	CE
Landa et al., (2011)	Interpersonal Synchrony (plus AEPS)	Both	10 h/wk		Caregiver & clinician	NIS
Lawton & Kasari, (2012b)	JASPER	Ind	In classroom; unable to determine specific amount	6 wks	Educator	DT
Mohammadzaheri et al., (2014)	PRT	Ind	2 60-min sessions/wk	3 mths	Clinician	ABA
Nefdt et al., (2010)	PRT (self-directed)	Ind	Unable to determine specific amount	1 wks	Caregiver	DT
Openden, (2005)	PRT	Ind	Unable to determine specific amount	4 days	Caregiver	DT
Rahman et al., (2016)	PASS	Ind	1-h session every 2 wks to train caregivers	6 mths	Caregiver	TAU
Schertz et al., (2013)	JAML	Ind	30 min/day w/caregiver & weekly teaching session w/intervention coordinator, caregiver, & child	4 – 12 mths	Caregiver	TAU
Schertz et al., (2018)	JAML	Ind	30 min/day w/caregivers & weekly 1-h teaching session w/intervention coordinator, caregiver, & child	32 wks	Caregiver	TAU
Schreibman & Stahmer, (2014)	PRT	Ind	2 2-h caregiver education sessions & 5 2-h in home sessions/wk for 15 wks, then 1 2-h education session & 2 2-h in-home sessions/wk for 8 wks	23 wks	Caregiver & clinician	PECS
Siller et al., (2013)	Focused Playtime Intervention	Ind	90 min of caregiver training/wk	12 wks	Caregiver	CE
Turner-Brown et al., (2019)	FITT	Ind	20 90-min in-home sessions & 4 caregiver-only clinic sessions	20 wks	Caregiver	TAU
Venker et al., (2012)	Adapted HMTW	Both	8–10 h of caregiver education, 1.5 h of individual sessions, & 12–14 h of small group sessions	~ 10 wks	Caregiver	DT
Vernon et al., (2019)	PRISM	Ind	8 h of 1:1 clinician intervention & 2 h of parent education with child/wk	6 mths	Caregiver & clinician	DT
Warreyn & Roeyers, (2014)	Training to promote imitation & JA	Ind	Two 30-min sessions/wk		Clinician	TAU
Wong, (2013)	JA intervention	Both	In classroom; unable to determine specific amount	4 weekly sessions	Educator	SP
Shared samples						
Aldred et al., (2004) sample	Communication-focused tx	Ind	Weekly sessions for 6 mths, then monthly sessions for 6 mths & 30 min daily of child with caregivers throughout	12 mths	Caregiver	TAU
Dawson et al., (2010) sample	ESDM	Ind	2 2-h sessions/day 5 days/wk for 2 years (mean of 15.2 h/wk) & mean of 16.3 h/wk of child w/caregiver	2 years	Caregiver & clinician	TAU
Green et al., (2010) sample	PACT	Ind	30 min daily home practice & 2 2-h clinic sessions/mth for 6 mths, then 6 monthly clinic sessions	12 mths	Caregiver	TAU
Kasari et al., (2006) sample	JA intervention	Ind	30 min/day during preschool program	5.5 wks	Clinician	SP & Control
Ingersoll, (2010) sample	RIT	Ind	3 1-h sessions/wk	10 wks	Clinician	TAU
Rogers et al., (2012) sample	ESDM	Ind	Weekly parent coaching for 3 mths, then 15 h/ wk in-home 1:1 intervention w/therapy assistants & 4 h of caregiver coaching for 24 mths	27 mths	Caregiver & clinician	TAU

Studies listed under “Independent Studies” include reports that do not share participants with any other included reports. Reports listed under “Shared Samples” share participants with at least one other included report. These reports are listed based on the earliest report

*ABA*  applied behavior analysis, *AEPS*  Assessment, Evaluation, and Programming System for Infants and Children, *ASAP*  advancing social-communication and play, *CDIT*  Child Directed Interaction Training, *CE * caregiver education, *Comp*  comparison, *DT * delayed treatment, *ESDM*  Early Start Denver Model, *FITT*  Family Implemented TEACCH for Toddlers, *HMTW*  Hanen More Than Words®, *hr*  hour, *Ind*.  individual, *JA*  joint attention, *JAML*  Joint Attention Mediated Learning, *JASPER*  Joint Attention, Symbolic Play, Engagement, and Regulation, *JE*  joint engagement, *min*  minute, *mth*  month, *NIS * Non-Interpersonal Synchrony, *PACT*  Preschool Autism Communication Trial, *PASS * Parent-mediated intervention for autism spectrum disorder in South Asia, *PECS*  Picture Exchange Communication System, *PRISM*  Pivotal Response Intervention for Social Motivation, *PRT * Pivotal Response Training/Treatment, *RIT *  Reciprocal Imitation Training, *SP*  Symbolic Play, *TAU*  treatment as usual, *wk*  week

**Table 4:** Effect size characteristics and outcome measures for included randomized controlled trials

	*n*	Prox./Dis		CB/PCB/GC	CME Risk	Mean *ES*	Outcome Measure(s)
Independent samples							
Boyd et al., (2018)	82	8/0		0/0/8	0	− 0.09	Observational coding of JA, requesting, and social interaction during ADOS
Carter et al., (2011)	28	6/6		2/3/7	5	− 0.07	ESCS for initiating JA and BR; MSEL Receptive and Expressive Communication; PIA Nonverbal Communication; PCFP weighted frequency of intentional communication; VABS Communication
Chang et al., (2016)	38	10/0		8/0/2	8	0.40	JA and BR during play session
Clionsky, (2012) & Ginn et al., (2017)	15	0/4		2/1/1	3	− 0.25	Child word count; PPVT-3; SRS Communication; total child verbalizations
Drew et al., (2002)	12	0/6		0/6/0	6	0.61	ADI RSI and Nonverbal Communication; ADOS Improved Spoken Language Classification; MCDI words understood, words said, and total gestures
Gengoux et al., (2019)	23	5/12		6/7/4	13	0.56	BOSCC Social Communication; CGI-Improvement; CGI-Severity; MCDI words produced; MSEL Expressive Language; PLS-5 Expressive Language; SLO imitative, prompted (verbally and nonverbally), spontaneous utterances; SRS-2 Social Communication; VABS Communication and Expressive Language
Goods et al., (2013)	5	4/2		2/0/4	0	0.51	Initiating JA and BR during ESCS and classroom observation; RDLS Verbal Communication and Expressive Language
Hardan et al., (2015)	25	5/9		5/8/1	13	0.39	CGI-Improvement; CGI-Severity; MCDI MLU and words said; PLS Expressive Communication; SLO imitative, prompted (verbally and nonverbally), and spontaneous utterances; VABS Communication, Expressive Language, and Receptive Language
Kaale et al., (2012)	34	3/0		1/1/1	2	0.25	Child-initiated higher order JA during ESCS, mother–child play, and teacher–child play
Kasari et al., (2010)	19	2/0		2/0/0	2	1.48	Initiating and responding to JA during caregiver-child interaction
Kasari et al., (2014)	51	2/0		0/0/2	0	− 0.14	ESCS Initiating JA
Kasari et al., (2015)	43	2/4		0/2/4	2	0.00	Initiating JA during parent–child interaction; RDLS Expressive Language and Receptive Language
Landa et al., (2011)	24	2/2		0/0/4	4	0.29	Initiating JA during CSBS; MSEL Expressive Language
Lawton & Kasari, (2012b)	9	15/0		10/0/5	10	0.67	Pointing, showing, giving, and looking during classroom observation, ESCS, and play interaction
Mohammadzaheri et al., (2014)	15	0/2		0/0/2	2	1.29	CCC and MLU
Nefdt et al., (2010)	13	1/0		1/0/0	1	0.89	Functional verbal utterances
Openden, (2005)	16	2/0		2/0/0	2	0.25	Functional verbal utterances and responsivity to opportunities for language
Rahman et al., (2016)	29	2/5		2/5/0	7	− 0.06	CSBS Social Composite and total weighted raw score; initiating communication acts; MCDI Expressive Language and Receptive Language; mutual shared attention; VABS Communication
Schertz et al., (2013)	11	4/3		4/1/2	5	0.78	MSEL Expressive Language and Receptive Language; PJAM initiating and responding to JA; VABS Communication
Schertz et al., (2018)	64	4/0		4/0/0	4	0.57	PJAM initiating and responding to JA
Schreibman & Stahmer, (2014)	20	0/6		0/4/2	4	− 0.33	MCDI words said; MSEL Expressive Language; VABS Communication
Siller et al., (2013)	34	0/2		0/0/2	0	0.97	MSEL Expressive Language
Turner-Brown et al., (2019)	32	0/3		0/2/1	2	− 0.19	MSEL Expressive Language; PIA Nonverbal Communication and Understanding
Venker et al., (2012)	7	3/0		3/0/0	3	0.11	Prompted, spontaneous verbal, and spontaneous nonverbal communication
Vernon et al., (2019)	12	0/9		0/1/8	9	0.87	EVT-2; MSEL Early Learning Composite, Expressive Language, and Receptive Language; PLS-5 Auditory Comprehension, Expressive Communication and Total Language; PPVT-4; VABS Communication
Warreyn & Roeyers, (2014)	18	9/0		0/0/9	9	0.50	Imitation (gestural, verbal, and symbolic) and JA (gaze following, initiating and responding to declarative JA, initiating requests, and reactions to ambiguous behavior) during examiner-child interaction
Wong, (2013)	18	2/0		2/0/0	2	0.41	Initiating and responding to JA during classroom observation
Shared Samples							
From Aldred et al., (2004) sample							
Aldred et al., (2004)	14	2/4		2/1/3	3	0.42	ADOS Reciprocal Social Interaction; child communication acts; child shared attention; MCDI words said and words understood; VABS Communication
Aldred et al., (2012)	14	0/1		0/0/1	0	0.76	ADOS Social Communication
From Dawson et al., 2010 sample							
Dawson et al., (2010)	24	0/4		0/2/2	2	0.41	MSEL Expressive Language and Receptive Language; VABS Communication
Dawson et al., (2012)	15	0/3		0/3/0	3	1.16	PDD-BI Expressive Social Communication, Receptive/Expression Social Communication, and Expressive Language
Sullivan, (2014)	24	0/5		0/0/5	1	0.37	MSEL Expressive Language and Receptive Language
Estes et al., (2015)	17	0/1		0/1/0	1	0.44	VABS Communication
From Green et al., (2010) sample							
Green et al., (2010)	74	2/8		2/3/5	5	0.15	ADOS-G Communication and Social Communication; child imitations and shared attention during parent–child interaction; CSBS; MCDI words said and words understood; PLS Auditory Comprehension and Expressive Communication; VABS Communication
Pickles et al., (2016)	77	4/2		4/1/1	5	0.27	CELF-4; child communication initiations; conversation turns; SCQ
From Kasari et al., (2006) sample							
Kasari et al., (2006)	20	22/0		0/0/22	0	0.26	Initiating and responding to JA during ESCS and mother–child interaction
Gulsrud et al., (2007)	17	4/2		6/0/0	6	0.02	Verbalizations; non-verbal gestures
Kasari et al., (2008)	20	24/12		0/0/36	0	0.16	Initiating responding to JA during ESCS and mother–child interaction; RDLS Expressive Language and Receptive Language
Kasari et al., (2012)	15	0/2		0/0/2	0	0.15	EVT
Lawton & Kasari, (2012a)	20	12/0		0/0/12	0	0.14	JA, shared positive affect, and utterances
From Ingersoll, (2010) sample							
Ingersoll, (2010)	11	1/0		0/0/1	0	1.38	Gesture imitation
Ingersoll, (2012)	14	0/1		0/0/1	0	0.83	ESCS initiating JA
From Rogers et al., (2012) sample							
Rogers et al., (2012)	49	1/5		0/5/1	5	− 0.11	JA; MCDI phrases understood, total gestures, words said, and words understood; VABS Communication
Rogers et al., (2019)	51	3/3		0/0/6	0	0.12	JA; MSEL Expressive Language and Receptive Language

*ADI*  Autism Diagnostic Interview (Lord et al., 1994), *ADOS * Autism Diagnostic Observation Schedule (Lord et al., 1999), *BOSCC * Brief Observation of Social Communication Change (Grzadzinski et al., 2016), *BR*  behavior regulation, *CB*  context-bound, *CCC*  Children’s Communication Checklist (Bishop, 2006), *CELF * Clinical Foundations of Language Fundamentals (Semel et al., 2006), *CGI*  Clinical Global Impressions Scale (Guy, 1976), *CME*  correlated measurement error, *CSBS*  Communication and Symbolic Behaviors Scale (Wetherby & Prizant, 2002), *Dis.*  distal, *ES*  effect size, *ESCS*  Early Social Communication Scales (Mundy et al., 2003), *EVT-2*  Expressive Vocabulary Test – Second Edition (Williams, 2007), *GC*  generalized characteristic, *JA*  joint attention, *MCDI*  MacArthur Communicative Development Inventory (Fenson et al., 1993), *MLU*  mean length of utterance, *MSEL*  Mullen Scales of Early Learning (Mullen, 1995), *PCB*  Potentially context-bound, *PCFP*  parent–child free play, *PDD-BI*  Pervasive Developmental Disorder – Behavior Inventory (Cohen et al., 2003), *PIA*  Parent Interview for Autism-Clinical Version (Stone et al., 2003), *PJAM*  Precursors of Joint Attention Measure (Schertz, 2005), *PLS*  Preschool Language Scale (Zimmerman et al., 2011), *PPVT*  Peabody Picture Vocabulary Test (Dunn & Dunn, 1997), *Prox*.  proximal, *RDLS * Reynell Developmental Language Scales (Reynell & Curwen, 1977), *RSI*  Reciprocal Social Interaction, *SCQ*  Social Communication Questionnaire (Rutter et al., 2003), *SLO*  structured laboratory observation, *SRS*  Social Responsiveness Scale (Constantino & Gruber, 2005), *SRS-2*  Social Responsiveness Scale—Second Edition (Constantino, 2012), *VABS * Vineland Adaptive Behavior Scales (Sparrow et al., 2005)

For the SCRD studies, Tables 5 and 6 display participant characteristics and intervention features. The studies included at least 143 unique participants with an average of 3.40 participants per study (*SD* = 1.17). Only participants who contributed data included for visual analysis or an effect size were included. The mean age of participants prior to intervention was 54.36 months (*SD* = 25.72). Table 7 displays effect size features (e.g., outcome measures and results) and visual analysis results.

**Table 5:** Participant characteristics for included single case research design studies

Reference	Pub	Loc	*n*	*M* age (mths)	T1 Developmental Level *M* (range)	ASD Severity *M* (range)
Becker, (2015)	No	USA*	4	41	MSEL Composite SS: 60 (49–80)	ADOS-2 severity: 8 (6–10)
Biller, (2018)	No	USA	4	51	MSEL VR AE: 25.5 (24–27)	NR
Calise et al., (2009)	Yes	USA*	1	150	NR	NR
Carpenter, (2003)	No	USA*	3	69	VABS Daily Living AE: 19 (*n* = 1)	NR
Christensen-Sandfort & Whinnery, (2013)	Yes	USA*	3	63	NR	CARS: 38.67 (32–45.5)
Coolican, (2010) & Coolican et al., (2010)	Mixed	Canada	3	52	IQ percentile (varied tests): 6 (< 1–16)	NR
Douglas et al., (2018)	Yes	USA	3	52	NR	NR
Dykstra et al., (2012)	Yes	USA	3	50	Leiter-R IQ: 71 (*n* = 1); MSEL AE: 43 & 46	ADOS Social Affect: 13.67 (12–17)
Gouvousis, (2012)	No	USA	3	49	NR	CARS-2: “mildly-moderately” to “severely”
Harjusola-Webb & Robbins, (2012)	Yes	USA	3	36	VABS-II Expressive AE: 11 (8–16)	CARS: 43 (40–47.5)
Higgins, (1999)	No	USA	3	46	VABS Cognitive AE: 11.67 (8–21)	NR
Hu et al., (2018)	Yes	China	3	64	Chinese WPPSI IQ: 107.67 (104–112)	Chinese CARS-2: 30.67 (30–32)
Huskens et al., (2012)	Yes	USA*	5	134	WISC-III NL IQ: 112.25 (105–121)	NR
Hwang & Hughes, (2000)	Yes	USA*	3	37	Uzgiris-Hunt: 8–12 or 12–18 m range	NR
Ingersoll et al., (2005)	Yes	USA*	3	36	Bayley or Brigance MA: 22 (19–25)	NR
Ingersoll et al., (2007)	Yes	USA*	5	41	Bayley or MSEL MA: 24.4 (16–31)	CARS: 37.9 (32–44.5)
Ingersoll, (2003) & Ingersoll & Schreibman, (2006)	Mixed	USA*	5	37	Bayley MA: 19.8 (15–29)	CARS: 35.4 (31.5–42); ADOS: 14.8 (13–16)
Ingersoll & Wainer, (2013)	Yes	USA*	5	48	Bayley NV MA: 29.2 (27–31)	NR
Jobin, (2013)	No	USA*	4	26	MSEL EL T-score: 24.5 (< 20–30)	NR
Laski et al., (1988)	Yes	USA*	8	78	MA: 3.56 (1.7–6.6)	NR
Law et al., (2018)	Yes	Singapore	3	42	VABS AB SS: 66.33 (63–70)	NR
Ma, (2010)	No	USA	3	49	NR	NR
Mancil, (2008) & Mancil et al., (2009)	Mixed	USA*	3	67	MA: 36.67 (29–49)	ADI reciprocal social interaction: 26 (26), communication: 17.33 (14–22), repetitive behaviors: 10 (10)
McGee et al., (1985)	Yes	USA	3	32	VABS AE: 3.77 (2.2–5.3)	NR
McGee & Daly, (2007)	Yes	USA*	3	59	Receptive vocabulary AE: 36 (30–> 48)	NR
Nichols, (2014)	No	USA*	4	42	NR	NR
Ogletree et al., (2012)	Yes	USA	1	84	NR	NR
Penney & Schwartz, (2019)	Yes	USA*	3	58	PPVT SS: 69 (55–96)	NR
Pierce, (1996) & Pierce & Schreibman, (1997)	Mixed	USA*	2	90	NV IQ: 63 (50–76)	NR
Randolph et al., (2011)	Yes	USA	3	60	VABS AB SS: 63.33 (55–71)	NR
Rocha et al., (2007)	Yes	USA	3	32	Bayley NV MA: 14.67 (12–18)	NR
Rollins et al., (2016)	Yes	USA	4	30	VB-MAPP milestones: 12 (8–15)	CARS: 44.63 (39.5–47); ADOS-2 total score: 22 (20–25)
Russell, (2014)	No	USA	3	57	NR	NR
Schertz & Odom, (2007)	Yes	USA*	2	24	HELP Cognitive AE: 15.75 (15–16.5)	CARS: 42.75 (40.5–45)
Sze, (2007)	No	USA	4	26	VABS Communication AE: 11.5 (9–14)	NR
Therrien & Light, (2018)	Yes	USA	3	52	PPVT SS: 60.67 (51–69)	CARS: 34.17 (30.5–36.5)
Thiemann & Goldstein, (2004)	Yes	USA*	5	91	Full scale IQ: 85.33 (47–117)	CARS: 33.7 (30–45.5)
Thiemann-Bourque et al., (2017)	Yes	USA*	3	54	PLS-4 Total SS: 50 (50)	"Severe"
Vernon et al., (2012)	Yes	USA*	3	38	VABS Communication AE: 18.33 (15–24)	NR
Vogler-Elias, (2009)	No	USA	3	54	P-TONI SS: 113 (106–120)	CARS: 36.33 (30–47.5)
Whalen, (2001) & Whalen & Schreibman, (2003)	Mixed	USA	4	50	Bayley MA: 18 (16–21)	CARS: 31.25 (30–32.5); GARS: 93.75 (90–105)
Zimmer, (2015)	No	USA	4	33	NR	NR

*AB*  adaptive behavior, *ADI*  Autism Diagnostic Interview (Lord et al., 1994), *ADOS*  Autism Diagnostic Observation Schedule (Lord et al., 1999), *AE*  age equivalent, *ASD*  autism spectrum disorder, *Bayley*  Bayley Scales of Infant & Toddler Development (Bayley, 1993), *CARS*  Childhood Autism Rating Scale (Schopler et al., 1993); Brigance = Brigance Inventory of Early Development-Revised (Brigance, 1991), *CARS-2*  Childhood Autism Rating Scale (Second Edition; Lu et al., 2004; Schopler et al., 2010), *EL*  expressive language, *GARS*  Gilliam Autism Rating Scale (Gilliam, 1995), *HELP*  Hawaii Early Learning Profile (Parks, 1992), *IQ*  intelligence quotient, *Leiter-R*  Leiter International Performance Scale–Revised (Roid & Miller, 1997), *Loc*.  location, *M*  mean, *MA*  mental age, *MSEL*  Mullen Scales of Early Learning (Mullen, 1995), *mths*  months, *NR*  not reported, *NV*  nonverbal, *PLS-4*  Preschool Language Scale – Fourth Edition (Zimmerman et al., 2002), *PPVT*  Peabody Picture Vocabulary Test (Dunn & Dunn, 1997), *P-TONI*  Primary Test of Nonverbal Intelligence (Ehrier & McGhee, 2008)**,** *Pub*.  published, *SS*  standard score, *T1 * Time 1 / prior to intervention, *USA * United States of America, *Uzgiris-Hunt*  Uzgiris-Hunt Ordinal Scales of Intellectual Development (Uzgiris-Hunt, 1975), *VABS*  Vineland Adaptive Behavior Scales (Sparrow et al., 1984), *VB-MAPP*  Verbal Behavior Milestones Assessment and Placement Program (Sundberg, 2008), *VR*  Visual Reception, *WISC-III NL*  Wechsler Intelligence Scale for Children – Netherlands (Kort et al., 2005), *WPPSI*  Wechsler Preschool & Primary Scale of Intelligence – Fourth Edition (Li et al., 2011; Wechsler, 2012)

**Table 6:** Intervention features of included single case research design studies

Study	Intervention	Ind. or Group	Amount of intervention	Duration	Interventionist
Becker, (2015)	CATE for Joint Attention Intervention	Ind	4 30-min sessions/wk	~ 30 sessions	Clinician
Biller, (2018)	NTS and SPS	Ind	2–3 30-min sessions/wk	6–7 mths	Clinician
Calise et al., (2009)	Contingent imitation	Ind	2 h/weekday	12 sessions	Clinician
Carpenter, (2003)	Naturalistic teaching strategies	Ind	10-min sessions (frequency NR)	18.7 sessions (mean)	Clinician
Christensen-Sandfort & Whinnery, (2013)	Milieu teaching strategies	Both	2 20-min sessions/wk	15–27 sessions	Educator
Coolican, (2010) & Coolican et al., (2010)	PRT	Ind	Varied (4–10 h/wk)	2 mths	Caregiver
Douglas et al., (2018)	Online communication partner training	Ind	Unable to determine	2–3 wks	Caregiver
Dykstra et al., (2012)	ASAP	Ind	40 + min 1:1 & 10–15 min group/wk	7.5–14 wks	Educator
Gouvousis, (2012)	PRT	Ind	Unable to determine	6–11 sessions	Educator
Harjusola-Webb & Robbins, (2012)	Naturalistic communication- promoting strategies	NR	20–40 min	6 mths	Educator
Higgins, (1999)	Semantic Pragmatic-Developmental intervention format	NR	1–3 30-min sessions/wk	10 sessions	Clinician
Hu et al., (2018)	Peer-Mediated LEGO® Play	Group	2 40-min sessions/wk	14–21 sessions	Peer
Huskens et al., (2012)	PRT	Ind	1–2 20-min sessions/wk	3–4 wks	Day treatment staff
Hwang & Hughes, (2000)	Social interactive training	Ind	2 10-min observations	30 wks	Clinician
Ingersoll et al., (2005)	DSP intervention	Ind	2 50-min sessions/wk	10 wks	Clinician
Ingersoll et al., (2007)	RIT	Ind	6 20-min sessions/wk (3 sessions/day)	10 wks	Clinician
Ingersoll, (2003) & Ingersoll & Schreibman, (2006)	RIT	Ind	8 20-min sessions/wk	10 wks	Clinician
Ingersoll & Wainer, (2013)	Project ImPACT	Ind	1–2 60-min sessions/wk	12 wks	Caregiver
Jobin, (2013)	PRT	Ind	3 45-min sessions/wk	12 wks	Clinician
Laski et al., (1988)	Natural Language Paradigm	Ind	60 min/wk w/caregiver & clinic visits	12 wks	Caregiver
Law et al., (2018)	Map4speech with natural language intervention	Ind	15 min/day 5 days/wk	Varied (mean = 6.7 wks)	Caregiver
Ma, (2010)	Naturalistic joint attention intervention	Ind	40 min/day	3–4 wks	Caregiver
Mancil, (2008) & Mancil et al., (2009)	Modified milieu therapy intervention	Ind	2–3 5-min sessions/wk	24–33 sessions	Caregiver
McGee et al., (1985)	Incidental teaching	Ind	~ 45 min/weekday	NR	Educator
McGee & Daly, (2007)	Incidental teaching	Group	5 min/weekday	17–43 sessions	Clinician or educator
Nichols, (2014)	Naturalistic Behavior Strategies	Ind	40–60 min	13–21 sessions	Clinician
Ogletree et al., (2012)	Milieu teaching sequence	Ind	2 10-min sessions 2–3 times/wk	15 sessions over 7 wks	Clinician
Penney & Schwartz, (2019)	RIT	Ind	100 min with caregiver; 30–40 min coaching (child present)	6–7 wks	Caregiver
Pierce, (1996) & Pierce & Schreibman, (1997)	PRT (peer-implemented)	Ind	1–2 10-min sessions/day	4–7 sessions	Peer
Randolph et al., (2011)	PRT	Ind	Unable to determine	5 wks	Caregiver & clinician
Rocha et al., (2007)	Joint attention parent training (DTT and PRT components)	Ind	3 20-min sessions/day 3 times/wk	6 wks	Caregiver & clinician
Rollins et al., (2016)	Pathways Early Autism Intervention	Ind	90-min session/wk	8–13 wks	Caregiver
Russell, (2014)	PRT	Ind	Unable to determine	1 wk	Caregiver
Schertz & Odom, (2007)	JAML	Ind	1 h per day	9–26 wks	Caregiver
Sze, (2007)	High-probability Behavioral Momentum Sequence	Ind	15–30 h	1–2 wks	Clinician
Therrien & Light, (2018)	Multicomponent intervention with AAC and turn-taking training	Dyad	1–3 5–20 min sessions/wk	5–9 sessions	Clinician
Thiemann & Goldstein, (2004)	Peer training and WTT	Ind	WTT 75–100 min; 10-min peer sessions (frequency NR)	21–37 sessions	Peer & clinician
Thiemann-Bourque et al., (2017)	Stay, Play, Talk	Dyad	2 sessions/wk (unknown length)	15–18 sessions	Peer
Vernon et al., (2012)	PRT plus embedded social interaction	Ind	3–5 1-h sessions/wk	16 sessions	Caregiver
Vogler-Elias, (2009)	Shared storybook reading instruction	Ind	5 min w/caregiver daily; 3 sessions w/caregiver & researcher/wk	12 sessions	Caregiver
Whalen, (2001) & Whalen & Schreibman, (2003)	Joint attention training	Ind	3 25-min sessions/day 3 days/wk	~ 10 wks	Clinician
Zimmer, (2015)	MITS	Ind	30-min sessions (frequency NR)	4 sessions	Caregiver

*AAC*  augmentative and alternative communication, *ASAP*  Advancing social-communication and play, *CATE*  Complexity Account of Treatment Efficacy, *DSP*  Developmental, Social–Pragmatic, *DTT * discrete trial training, *hr*  hour, *Ind*. Individual, *ImPACT*  IMproving Parents As Communication Teachers, *JAML*  Joint Attention Mediated Learning, *min * minute, *MITS*  Meaningful Interactions Through Storybooks, *mth*  month, *NR*  not reported, *NTS*  natural teaching strategies, *PRT*  Pivotal Response Training/Treatment, *RIT*  Reciprocal Imitation Training, *SPS * speech production strategies, *wk*  week, *WTT * written text treatment

**Table 7:** Visual analysis and effect size results for included single case research design studies

	Design	Quality	Outcome measure(s)	Demos. of FR	FR Opps	Strength of evidence	# of *ES*s	Mean *ES*
Becker, (2015)	MB-P	w/o res	Initiating & responding to points, gives, & gaze shifts	0	7	No	7	0.31
Biller, (2018)	MP-P	w/res	Production score	1	1	Strong	1	2.44
Calise et al., (2009)	ABABAB	w/res	Vocalizations per minute	1	1	Strong	CNC	CNC
Carpenter, (2003)	MB-P	w/o res	Coordinated JA; spontaneous speech & verbalizations	CNA	CNA	N/A	4	0.88
Christensen-Sandfort & Whinnery, (2013)	MB-P	w/res	Spontaneous responses	1	2	Strong: 1; No: 1	2	1.16
Coolican, (2010) & Coolican et al., (2010)	MB-P	w/res	Functional verbal utterances	CNA	CNA	N/A	1	0.85
Douglas et al., (2018)	MP-P	w/res	Child communication turns	0	1	No	1	0.28
Dykstra et al., (2012)	MB-P	w/o res	Social communication	0	2	No	2	0.70
Gouvousis, (2012)	MB-P	w/o res	Spontaneous, prompted, & echoic words & phrases	2	6	Strong: 2; No: 4	6	1.09
Harjusola-Webb & Robbins, (2012)	MB-P	w/o res	Expressive communication	1	1	Strong	1	2.20
Higgins, (1999)	AATD	w/o res	Verbal & nonverbal (semiotic) behaviors	2	6	Strong: 2; No: 4	CNC	CNC
Hu et al., (2018)	ABAB	w/o res	Social initiations & responses	2	2	Strong	CNC	CNC
Huskens et al., (2012)	MB-P	w/res	Initiatives; spontaneous initiatives	1	2	Moderate: 1; No: 1	2	0.50
Hwang & Hughes, (2000)	MB-P	w/res	JA	1	1	Strong	1	2.61
Ingersoll et al., (2005)	MB-P	w/res	Spontaneous expressive language	CNA	CNA	N/A	1	1.17
Ingersoll et al., (2007)	MB-P	w/o res	Imitation & spontaneous use of gestures	2	4	Strong: 2; No: 2	4	1.36
Ingersoll, (2003) & Ingersoll & Schreibman, (2006)	MB-P	w/o res	Imitated & spontaneous language; coordinated JA; coordinated JA w/PA	CNA	CNA	N/A	5	0.88
Ingersoll & Wainer, (2013)	MB-P	w/o res	Spontaneous language	0	1	No	1	0.39
Jobin, (2013)	AATD	w/o res	Acquired & generalized receptive & expressive language	6	16	Strong: 6; No: 10	CNC	CNC
Laski et al., (1988)	MB-P	w/res	Child vocalizations	1	3	Strong: 1; No: 2	3	1.47
Law et al., (2018)	MB-P	w/o res	Prompted utterances & points	0	1	No	1	0.25
Ma, (2010)	MB-P	w/res	Independent initiating JA	1	1	Strong	1	1.33
Mancil, (2008) & Mancil et al., (2009)	MB-P	w/o res	Communication responses	1	1	Strong	1	2.21
McGee et al., (1985)	MB-P	w/o res	% correct on acquisition probe	1	1	Strong	CNC	CNC
McGee & Daly, (2007)	MB-P	w/o res	Conversational phrases per min	1	1	Strong	1	0.70
Nichols, (2014)	MB-P	w/o res	Mands (total, unprompted, with social engagement)	3	3	Strong	3	3.05
Ogletree et al., (2012)	MB-B	w/res	Trained exchanges	0	1	No	CNC	CNC
Penney & Schwartz, (2019)	MB-P	w/res	Spontaneous imitation	0	1	No	1	0.44
Pierce, (1996) & Pierce & Schreibman, (1997)	MB-Peer	w/res	Initiations	0	2	No	CNC	CNC
Randolph et al., (2011)	MB-P	w/res	Verbal responses; child initiations	0	2	No	2	0.26
Rocha et al., (2007)	MB-P	w/res	Responding to JA bids (in sessions & generalization probe)	1	2	Strong: 1; No: 1	2	1.74
Rollins et al., (2016)	MB-P	w/o res	Verbal reciprocity	1*	1	Moderate	1	1.95
Russell, (2014)	MB-P	w/o res	Functional verbal utterances	0	1	No	1	0.07
Schertz & Odom, (2007)	MB-P	w/o res	Turn-taking; initiating & responding to JA	1	2	Strong: 1; No: 1	CNC	CNC
Sze, (2007)	MB-P	w/o res	Functional responding to target word stimuli; number of words; vocabulary diversity	3	3	Strong	3	3.85
Therrien & Light, (2018)	MB-P	w/o res	Symbolic turns	1	1	Strong	1	2.56
Thiemann & Goldstein, (2004)	MB-P & MB-B	w/o res	Social communication skills	5	7	Strong: 5; No: 2	2	1.22
Thiemann-Bourque et al., (2017)	MP-P	w/o res	Spontaneous communication acts directed to peers	1	1	Strong	1	1.05
Vernon et al., (2012)	MB-P	w/o res	Verbal initiations	1	1	Strong	1	1.23
Vogler-Elias, (2009)	MB-P	w/o res	Number of different words	0	2	No	1	0.11
Whalen, (2001) & Whalen & Schreibman, (2003)	MB-P	w/res	Following & using gaze shift & pointing	CNA	CNA	N/A	3	6.63
Zimmer, (2015)	MP-P	w/res	JA behaviors per minute	1	1	Strong	1	4.59

*Three demonstrations of an effect with one demonstration of a non-effect, but one participant who showed an effect began intervention at the same time as the participant who showed a non-effect; *AATD*  adapted alternating treatments design, *CNA*  could not analyze, *CNC*  could not calculate, *ES*  effect size, *Demos. of FR*  number of demonstrations of a functional relation, *FR Opps*.  opportunities to show a functional relation, *JA*  joint attention, *MB-B*  multiple baseline across behaviors, *MB-P*  multiple baseline across participants, *N/A*  not applicable, *PA*  positive affect, *w/o res*  without reservations based on What Works Clearinghouse standards (What Works Clearinghouse, 2016), *w/res*  with reservations based on What Works Clearinghouse standards (What Works Clearinghouse, 2016)

### Quality Indicators

For the RCTs, overall risk of bias was judged to be high for 25 studies, moderate for 7 studies, and low for only 1 study. It should be noted that a “high” risk of bias rating for any category results in an overall risk of bias rating of “high”. See Tables 8 and 9 for details. Many studies were noted to be at risk for CME which resulted in high risk of bias for “Measurement of outcome.” Only seven of the RCTs provided sufficient information to determine whether there were deviations from the intended intervention (a component of “Deviations from intended interventions”). Of those, three indicated probable risk of bias. Studies were judged to deviate from the intended intervention if the mean or median procedural fidelity value was below 80%. “Sufficient information” required that procedural fidelity data to be drawn from at least 20% of sessions or participants. Similar gaps in reporting of outcome measure reliability were also observed, as shown in Table 9. The high number of studies without sufficient information about procedural fidelity and reliability reveals an area of need for improving the quality of available studies. It inhibits quantitative analysis of the influence of procedural fidelity and reliability on intervention effects. No studies were at high risk of bias for the randomization process and only three were at high risk for deviations from the intended interventions.

**Table 8:**
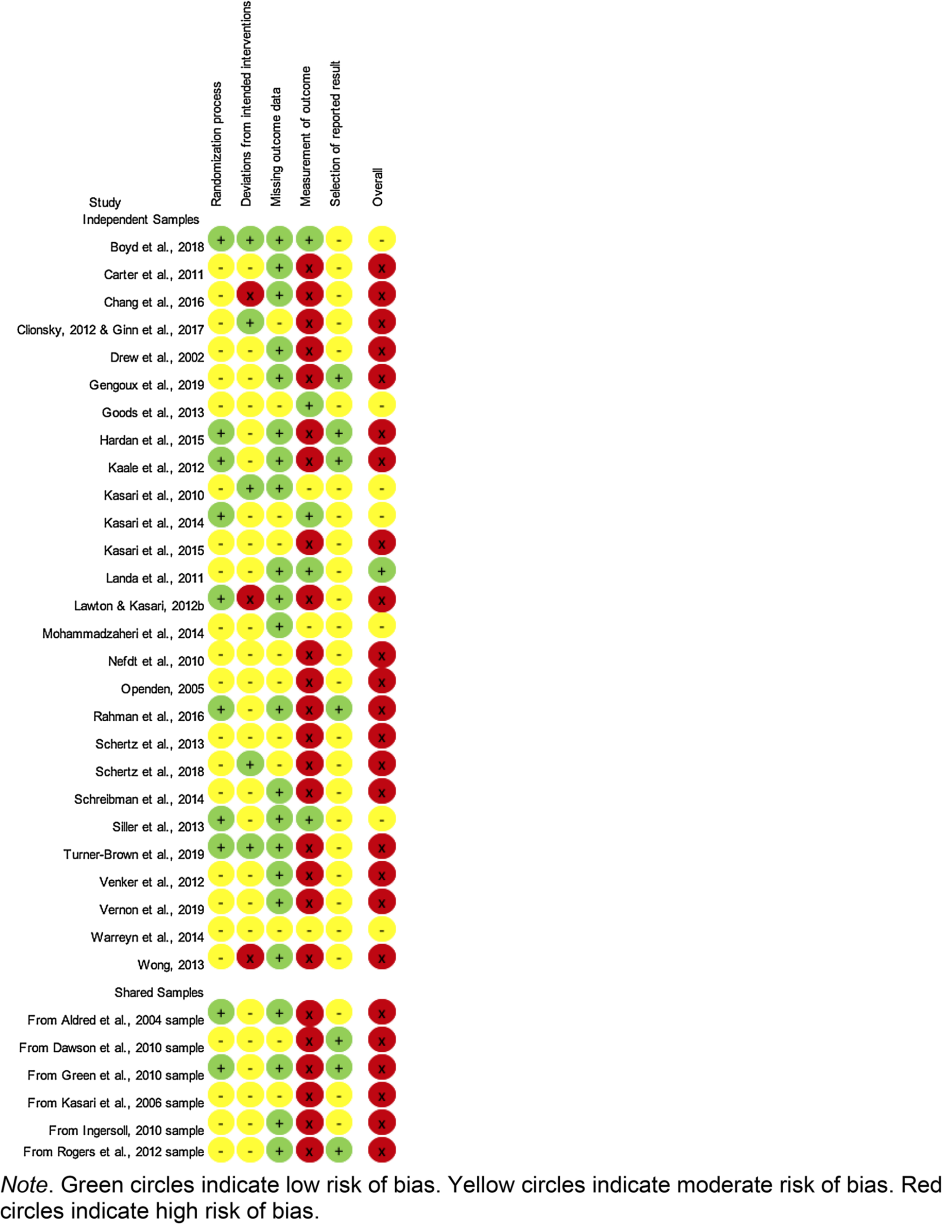
Risk of bias for included randomized controlled trials

For the SCRD studies, only three studies included at least one distal outcome measure (Carpenter, 2003; Ingersoll & Wainer, 2003, 2013). All outcome measures were context-bound and at risk for CME. The high proportion of proximal and context-bound outcome measures is consistent with Yoder et al. (2013). For study quality, 25 of the 47 included reports met quality standards without reservations. As shown in Fig. 1, 44 reports that otherwise would have been included were excluded due to failing to meet quality standards (listed in Supplementary Information 2). Twenty-three SCRDs provided some type of summary value for procedural fidelity of the interventionist (see Table 10). Nine additional SCRD studies provided fidelity data for the interventionists, but not in a summative form (e.g., graphically or narrative description). However, only two studies (Randolph et al., 2011; Vogler-Elias, 2009) reported procedural fidelity data for the trainers (e.g., a trainer who taught a caregiver to implement the intervention). The ten remaining SCRD studies did not report procedural fidelity data. Similar to the RCTs, the gaps in reporting of procedural fidelity reveal an area of need for improving the quality of available studies. Relative to procedural fidelity data, the SCRD studies more consistently reported interobserver agreement (IOA) data for the outcome measure. Only one study omitted IOA data, revealing an area of strength for the included studies.

**Table 9:** Procedural fidelity bias rating and outcome measure reliability for included randomized controlled trials

	PF Bias Rating	Outcome Measure Reliability		
		Type	Value	% of sessions
Independent samples				
Boyd et al., (2018)	NI	ICC	.93 (across all included OMs from ADOS)	20%
Carter et al., (2011)	NI	ICC	ESCS: .96; PCFP: .98; other OMs: NR	Approximately 20%
Chang et al., (2016)	PY	ICC	.92 (across all included play session OMs)	NR
Clionsky, (2012) & Ginn et al., (2017)	PN	Kappa	Total child verbalizations: .66; word count: .13; other OMs: NR	27%
Drew et al., (2002)	NI	NR	NR	NR
Gengoux et al., (2019)	NI	ICC	SLO: .94; BOSCC: .86; other OMs: NR	30%
Goods et al., (2013)	NI	ICC	ESCS: .85; other OMs: NR	NR
Hardan et al., (2015)	NI	ICC	SLO: .96; other OMs: NR	> 33%
Kaale et al., (2012)	NI	ICC	ESCS: .68; teacher–child play: .62; mother–child play: .79	16–22% depending on OM
Kasari et al., (2010)	PN	ICC	.78	20%
Kasari et al., (2014)	NI	ICC	.80	NR
Kasari et al., (2015)	NI	ICC	IJA: .97; RDLS: NR	NR
Landa et al., (2011)	NI	ICC	IJA: .95; MSEL: NR	20%
Lawton & Kasari, (2012b)	PY	ICC	Classroom observation: .77; ESCS: .89; play observation: .85	20%
Mohammadzaheri et al., (2014)	NI	Percent agreement	CCC: .99; MLU: NR	40%
Nefdt et al., (2010)	NI	Point-by-point agreement	93%	35%
Openden, (2005)	NI	Point-by-point agreement	Functional verbal utterances: 91%; responsivity to opportunities: 93%	> 33%
Rahman et al., (2016)	NI	ICC	DCMA: 85; other OMs: NR	20%
Schertz et al., (2013)	NI	Kappa	PJAM: .80; other OMs: NR	25%
Schertz et al., (2018)	PN	NR	NR	NR
Schreibman & Stahmer, (2014)	NI	NR	NR	NR
Siller et al., (2013)	NI	NR	NR	NR
Turner-Brown et al., (2019)	PN	NR	NR	NR
Venker et al., (2012)	NI	ICC	.96 across all OMs	20%
Vernon et al., (2019)	NI	NR	NR	NR
Warreyn & Roeyers, (2014)	NI	Kappa	JA: .60–.93; imitation: .72–.85	15%
Wong, (2013)	PY	ICC	.86	NR
Shared samples				
From Aldred et al., (2004)sample	NI	Percent agreement / kappa	Parent–child interaction: 90% / .89; other OMs: NR	25%
From Dawson et al., (2010) sample	NI	NR	NR	NR
From Green et al., (2010) sample	NI	ICC	Parent–child interaction: .59; ADOS standard scoring .79; ADOS modified scoring: .83; child initiations: .8; conversation turns: .9; other OMs: NR	Parent–child interaction: 14%; ADOS: 10%; child initiations & conversational turns: 22 ratings
From Kasari et al., (2006) sample	NI	ESCS: Kappa / ICC; mother–child interaction: ICC; JA probe: Kappa	ESCS: .79 / .81; mother–child interaction: .85; JA probe (Gulsrud et al., 2007): .88; RDLS & EVT: NR	ESCS: 20%; mother–child interaction: NR; JA probe: 25%
From Ingersoll, (2010) sample	NI	MIS and UIA: Kappa; ESCS: small/large	MIS: .93; UIA: .84; ESCS: 80%	25%
From Rogers et al., (2012) sample	NI	NR	NR	NR

The PF Bias Rating is based on the Revised Cochrane risk-of-bias tool for randomized trials (Higgins et al., 2019). Point-by-point agreement is the number of agreements divided by the total number of agreements and disagreements multiplied by 100. *ADOS * Autism Diagnostic Observation Schedule (Lord et al., 1999), *BOSCC * Brief Observation of Social Communication Change (Grzadzinski et al., 2016), *CCC*  Children’s Communication Checklist (Bishop, 2006), *DCMA * Dyadic Communication Measure for Autism, *ESCS*  Early Social Communication Scales (Mundy et al., 2003), *EVT*  Expressive Vocabulary Test (Williams, 1997), *ICC * intraclass correlation coefficient, *IJA * initiating joint attention, *JA*  joint attention, *MIS*  Motor Imitation Scale (Stone et al., 1997), *MLU*  mean length of utterance, *MSEL*  Mullen Scales of Early Learning (Mullen, 1995), *NI*  No information or insufficient information, *NR*  not reported, *OMs * outcome measures, *PCFP*  parent–child free play, *PF * procedural fidelity, *PJAM*  Precursors of Joint Attention Measure (Schertz, 2005), *PN*  probably no (not biased), *PY*  probably yes (biased), *RDLS*  Reynell Developmental Language Scales (Reynell & Curwen, 1997), *SLO*  structured laboratory observation, *UIA*  Unstructured Imitation Assessment

### Effect Size

We reject the null hypothesis that there is no effect of interventions using responsivity strategies on prelinguistic and language skills of children with ASD for the RCTs and SCRD studies (research question 1). For the RCTs, the mean standardized group difference is *g* = 0.36, 95% CI [0.21, 0.51], which is a moderate effect size. No variation in the weighted mean effect sizes were observed when we varied the *p* value in Stata at 0.1 increments from 0.0 to 0.9.

For the SCRD studies, the mean BC-SMD = 1.20, 95% CI [0.87, 1.54], which is large. No variation in the weighted mean effect sizes were observed when we varied the *p* value in Stata at 0.1 increments from 0.0 to 0.9. The difference in mean effect size between the RCTs and SCRDs may be due to methodological differences between group and SCRD studies. Thus, the effect sizes are not directly comparable between the RCTs and the SCRD studies. Relatively large effect sizes are easier to detect through visual analysis and may explain the publication bias toward studies with larger effects for SCRD studies (Shadish et al., 2015, 2016). As described in the Moderator Analyses section, we did identify evidence of publication bias. Other meta-analyses that combine group and SCRD studies have also reported relatively larger mean effect sizes for SCRD studies (Barton et al., 2017). Based on visual analysis of the SCRD studies, 41 graphs (45%) showed strong evidence, two (2%) showed moderate evidence, and 48 (53%) showed no evidence of a functional relation between the intervention with responsivity strategies and child prelinguistic and/or language skills. Opportunities to show a functional relation that showed strong evidence had a mean BC-SMD of 2.34 (*SD* = 2.18, range: 0.56 – 5.36). Those that showed no evidence had a mean BC-SMD of 0.67 (*SD* = 0.49, range: -0.20 – 2.69).

### Moderator Analyses

The moderator analyses address our second research question about whether particular study features account for the observed heterogeneity. RCTs and SCRD studies were analyzed separately.

**Fig. 2:**
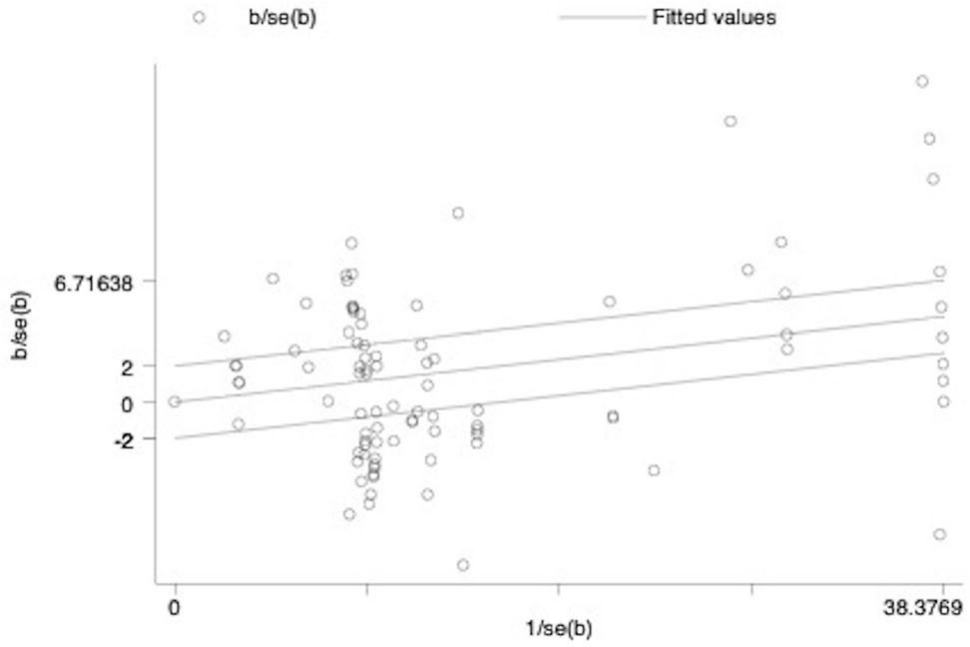
Galbraith plot for included randomized controlled trials

**Fig. 3:**
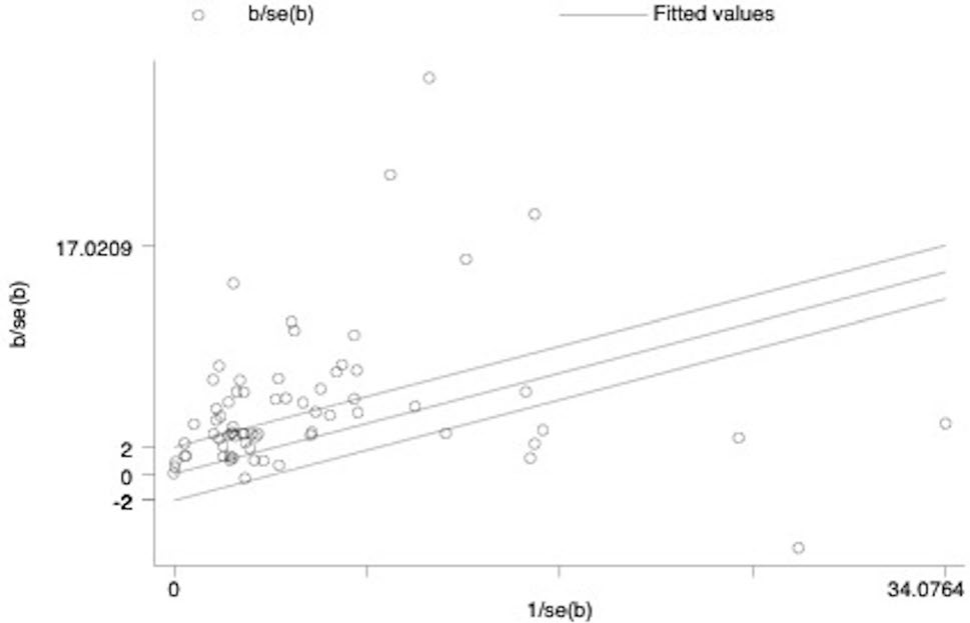
Galbraith plot for included single case research design studies

The Galbraith plots (Figs. 2 and 3) and τ^2^ values (0.19 and 0.67 for RCTs and SRCD designs, respectively) all provide evidence of substantial heterogeneity. We define heterogeneity as variation in estimated ‘true effects’ (Borenstein et al., 2009). This variation is differentiated from that due to spurious error in the computation of τ^2^ by considering the ratio of observed to expected variation across studies. The results show that there is notable dispersion of the effect sizes that is assumed to be real rather than spurious error. The larger τ^2^ value for SRCD studies than RCTs indicates greater dispersion in true effects for the SRCD studies than the RCTs. The Galbraith plot, which is an alternative to the forest plot for meta-analyses with a large number of effect sizes, displays more precise estimates further from the origin. The large number of effect sizes outside of the two parallel outer lines that represent that 95% confidence interval indicates substantial heterogeneity (Anzures-Cabrera & Higgins, 2010).

#### RCTs

For the RCTs, six moderator analyses were planned (i.e., interventionist, time in intervention, proximity, boundedness, risk for CME, and publication status). Context-bound outcomes exhibited a larger mean effect size (*p* < 0.05; *g* = 0.47) than generalized or potentially context-bound outcomes combined (*g* = 0.24). These results indicate the participants exhibited larger changes in behaviors that are measured in situations very similar to the treatment sessions (i.e., context-bound) than those measured in situations that vary from the treatment context in setting, materials, and/or communication partner. No other moderator analyses yielded significant results. Due to missing details in the included reports, time in intervention could only be extracted for 18 of the 33 RCTs. As a result, the degrees of freedom were too low to complete the analysis for time in intervention. Only a few studies that included caregivers as the interventionists reported the time caregivers spent conducting the intervention (Clionsky, 2012; Dawson et al., 2010; Green et al., 2010). As an alternative intensity variable, we tested time in intervention in weeks. However, even with more studies providing such information, the degrees of freedom were still too low (i.e., < 4) for a trustworthy result. Similarly, due to studies rarely being unpublished (i.e., four effect sizes from two studies), the degrees of freedom for this analysis were too low to interpret.

Table 11 displays results by subgroups to inform decisions regarding which moderators may warrant additional investigation. Of note, the mean effect size was greater than zero for effect sizes at risk for CME (*g* = 0.39), but not for those free from CME risk (*g* = 0.12). Except for the caregiver only subgroup, the relatively low number of studies in the interventionist subgroups resulted in low degrees of freedom and should be interpreted with caution.

**Table 10:** Procedural fidelity for interventionist and interobserver agreement for included single case research design studies

	Interventionist procedural fidelity		Interobserver agreement		
	Value	% of sessions	Type	Value	% of sessions
Becker, (2015)	No summary score; describes selected intervention steps	26	ICC	.99	24
Biller, (2018)	“averaged at least 90% across the six strategies" (p. 52)	25	Point-by-point agreement	90%	25
Calise et al., (2009)	NR	NR	Frequency ratio	95%	33 (phases 5 & 6)
Carpenter, (2003)	NR	NR	Point-by-point agreement	87%	33–39
Christensen-Sandfort & Whinnery, (2013)	100%	7–13	Small/large	94%	28
Coolican, (2010) & Coolican et al., (2010)	No summary score; 0, 5, and 4 of 8 parents met 75% criteria at pre, post, and follow-up phases	> 20	Point-by-point agreement / kappa	86% / .85	30
Douglas et al., (2018)	Not assessed	N/A	Point-by-point agreement	97%	21
Dykstra et al., (2012)	91%	15–20	Point-by-point agreement	95–98%	19–21
Gouvousis, (2012)	Teacher training phase: 78%; PRT treatment phase: 85%	100	Point-by-point agreement	93%	40
Harjusola-Webb & Robbins, (2012)	NR	NR	Point-by-point agreement	89%	20
Higgins, (1999)	NR	NR	Point-by-point agreement	97%	Total of 12 2-min segments
Hu et al., (2018)	100%	36	Point-by-point agreement	88–91%	33–40
Huskens et al., (2012)	97%	33	Point-by-point agreement	88–98%	33
Hwang & Hughes, (2000)	No summary value; frequency of training strategy use in Table 5	100	Point-by-point agreement / kappa	86%/ .79	28
Ingersoll et al., (2005)	.90	10	Kappa	.61	25
Ingersoll et al., (2007)	.96	10	Kappa	.66–.73	25
Ingersoll, (2003) & Ingersoll & Schreibman, (2006)	.96	10	Kappa	.73–.94	33
Ingersoll & Wainer, (2013)	No summary value; graphed	100	ICC	.93	25
Jobin, (2013)	.99	33	Point-by-point agreement	91%	33
Laski et al., (1988)	NR	NR	Point-by-point agreement	87–96%	49
Law et al., (2018)	83–97% depending on phase	100	Percentage	85–97%	33
Ma, (2010)	98% (averaged across all phases)	17–100	Point-by-point agreement	92–100%	17–100
Mancil, (2008) & Mancil et al., (2009)	92%	100	Point-by-point agreement / kappa	95% / .90	50–100
McGee et al., (1985)	NR	NR	Point-by-point agreement	99%	21
McGee & Daly, (2007)	No summary value provided	NR	Occurrence agreement	90%	25
Nichols, (2014)	100%	30	Small/large	95%	25
Ogletree et al., (2012)	100%	< 20	Point-by-point agreement	83%	30% of opportunities
Penney & Schwartz, (2019)	87%	~ 20	Point-by-point agreement	95%	22
Pierce, (1996) & Pierce & Schreibman, (1997)	NR	NR	Point-by-point agreement	92%	33
Randolph et al., (2011)	2 of 3 caregivers reached the 80% criterion during intervention	100	Point-by-point agreement	94%	27–50
Rocha et al., (2007)	91%	25—> 29	Point-by-point agreement / kappa	84% / .94	> 33
Rollins et al., (2016)	85%	100	Kappa	99%	20
Russell, (2014)	46%	NR	Pearson's correlation	.99	25
Schertz & Odom, (2007)	No summary value; see Table 2	NR	Kappa	.83	25
Sze, (2007)	100%	33	Point-by-point agreement	95–98%	33
Therrien & Light, (2018)	99%	30	Point-by-point agreement	94%	30
Thiemann & Goldstein, (2004)	> 80%	25	Point-by-point agreement	90%	33
Thiemann-Bourque et al., (2017)	92%	41	Point-by-point agreement	92%	30
Vernon et al., (2012)	93%	50	Point-by-point agreement / kappa	90% / .77	33
Vogler-Elias, (2009)	NR	NR	NR	NR	NR
Whalen, (2001) & Whalen & Schreibman, (2003)	93–100%	10	Point-by-point agreement / kappa	> 80% / .87	33
Zimmer, (2015)	NR	NR	Point-by-point agreement	88%	33

Point-by-point agreement is the number of agreements divided by the total number of agreements and disagreements multiplied by 100. *NR * not reported

We calculated the correlations between each of the tested moderators to evaluate how distinct each moderator is from the others. Of all the pairs, only three exceeded *r* = 0.30: proximity of the outcome measure and time in intervention in weeks (*r* = 0.34), risk for CME and boundedness (*r* = 0.68), and time in intervention in weeks and time in intervention in hours (*r* = 0.75). Distal outcome measures were more likely to be used for studies of relatively longer duration. Studies not at risk for CME were more likely to use generalized outcome measures. The relatively high correlation between the time in intervention in weeks and time in intervention in hours is expected; time in intervention in weeks was derived as an alternative to time in intervention in hours to address missing data in the included reports.

**Fig. 4:**
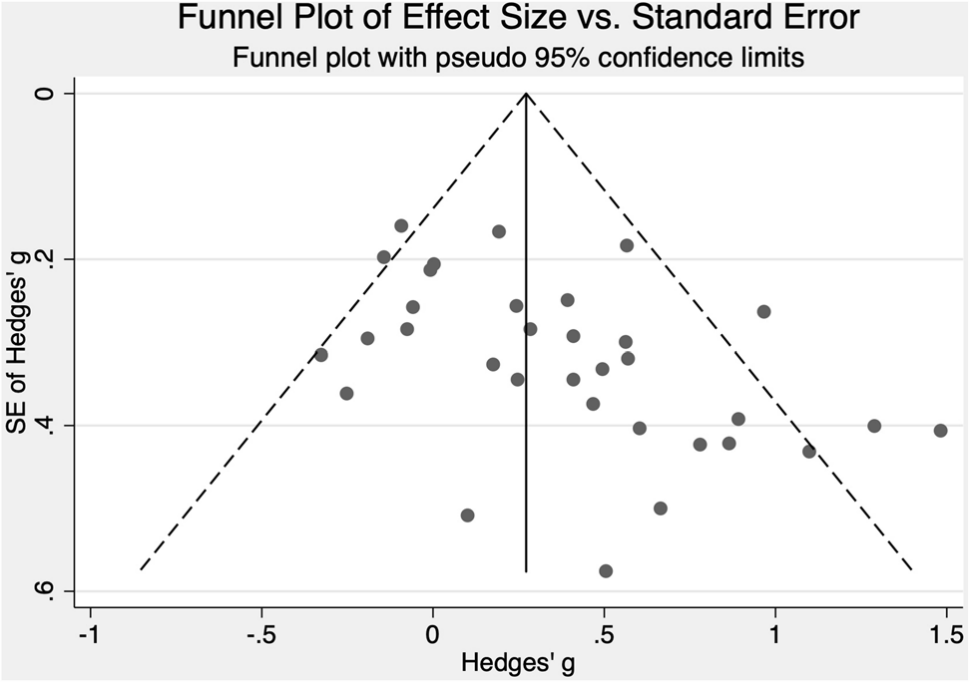
Funnel plot of effect size (Hedges’ g) versus standard error for included randomized controlled trials

**Fig. 5:**
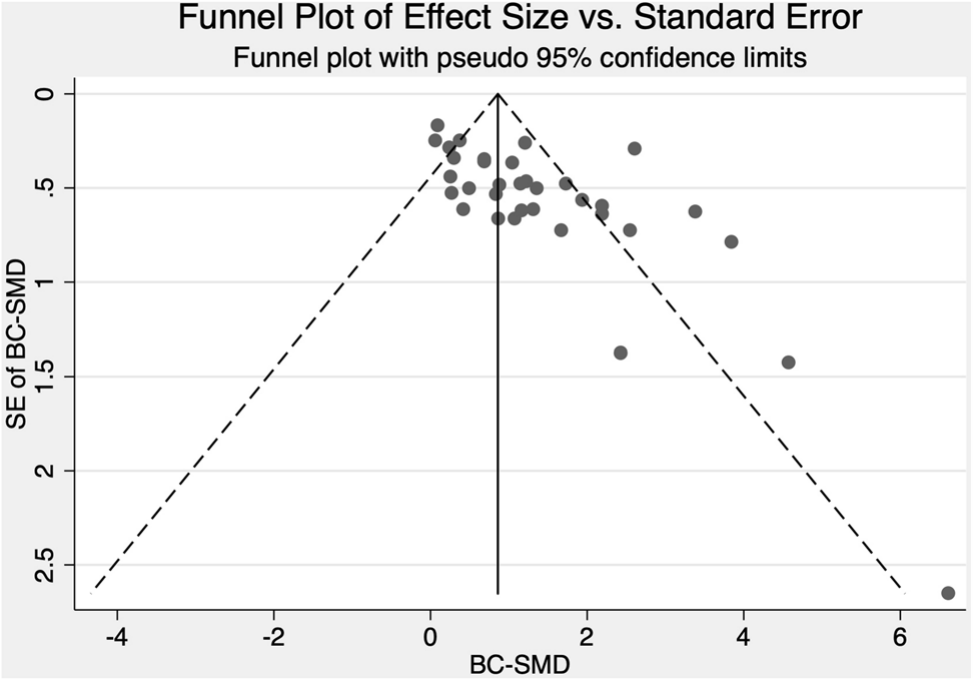
Funnel plot of effect size (between-case standardized mean difference [BC-SMD]) versus standard error for included single case research design studies

A publication bias was not detected via the moderator analysis. However, the Egger’s test suggests publication bias against small studies with negative results (Fig. 4; *p* < 0.01). The moderator analysis for publication bias was likely limited by the relatively low number of effect sizes (i.e., four) reported from unpublished reports (Fig. 5).

#### SCRD Studies

For the SCRD studies, three moderator analyses (i.e., interventionist, time in intervention, and publication bias) were completed. The other moderators tested for the RCTs did not have enough variation across the SCRD studies. All of the effect sizes were at risk for CME and used context-bound outcome measures. Only 7 effect sizes included distal outcome measures. For time in intervention, only 13 studies reported the necessary details. None of the moderator effects were significant. See Table 12 for moderator analyses by subgroup. No correlations between moderators exceeded *r* = 0.4. We completed a follow-up analysis comparing only studies implemented by a caregiver alone or a clinician alone, which were the two types of interventionists with sufficient degrees of freedom for reliable results. Effect sizes for interventions implemented by caregivers only had a mean effect size of 0.81 versus 1.90 for those implemented by clinicians only. Results approached, but did not reach, statistical significance (*p* = 0.06). Given the magnitude of difference in mean effect sizes and identified differences in prior meta-analyses, the role of interventionists warrants continued evaluation in the future, especially as the number of relevant primary studies increases. A publication bias was not detected via the moderator analysis. However, like the RCT analysis, the Egger’s test suggests publication bias against small studies with negative results (*p* < 0.001).

**Table 11:** Moderator analysis results by subgroup for included randomized controlled trials

Subgroup	*n*	*df*	*g*	95% CI
Overall weighted mean effect size	294	30.73	0.36	[0.21, 0.51]
Interventionist				
Caregivers	104	16.49	0.30	[0.07, 0.53]
Clinicians	97	3.92	–	–
Caregivers and clinicians	55	4.83	0.31	[-0.09, 0.71]
Educators	38	3.51	–	–
Proximity of outcome measure				
Proximal	166	22.72	0.40	[0.24, 0.55]
Distal	128	19.12	0.28	[0.08, 0.47]
Boundedness of outcome measure				
Context-bound	70	17.44	0.47	[0.27, 0.67]
Potentially context-bound	57	13.67	0.21	[0.03, 0.39]
Generalized	167	22.21	0.19	[0.03, 0.36]
Correlated measurement error				
At risk	145	25.29	0.39	[0.22, 0.56]
Not at risk	149	18.77	0.12	[-.03, 0.27]
Published	290	29.77	0.36	[0.21, 0.52]
Not published	4	1.00	–	–

When *df* is less than 4, results are omitted because they should be interpreted with caution. *df*  degrees of freedom, *n*  number of effect sizes, *g*  mean standardized group difference

**Table 12:** Moderator analysis results by subgroup for included single case research design studies

Subgroup	*n*	*df*	BC-SMD	95% CI
Overall weighted mean effect size	69	30.49	1.20	[0.87, 1.54]
Interventionist				
Caregivers	15	10.37	0.81	[0.32, 1.29]
Clinicians	33	9.01	1.90	[0.99, 2.80]
Caregivers and clinicians	4	1.00	–	–
Educators	12	3.54	1.06	[0.32, 1.79]
Published	40	21.37	1.16	[0.84, 1.48]
Not published	29	9.15	1.32	[0.35, 2.29]

When *df* is less than 4, results are omitted because they should be interpreted with caution. *BC-SMD* between-case standardized mean difference, *df*  degrees of freedom, *n*  number of effect sizes

## Discussion

### Summary of Evidence

Based on 294 effect sizes from 33 RCTs and 69 effect sizes from 34 SCRD studies that included a total of 1040 participants, the weighted mean effect size of the effect of interventions using responsivity intervention strategies on child prelinguistic and language outcomes is moderate to large. The identified mean effect size (*g* = 0.36) is somewhat larger than that identified by Hampton and Kaiser (2016; *g* = 0.26) and Sandbank et al., (2020b; *g* = 0.13 for receptive language; *g* = 0.18 for expressive language;), which evaluated a wider variety of interventions on language outcomes. Visual analysis of 91 opportunities to demonstrate a functional relation from 37 SCRD studies provided somewhat weaker support, characterized by 45% of opportunities showing strong support, 2% showing moderate support, and 53% showing no support for the interventions improving child prelinguistic and/or language outcomes. Thus, heterogeneity in results is apparent through the effect sizes and visual analysis. Although the mean effect size for the SCRD studies was large, a nearly even split between “strong” and “no” evidence offers reason for caution in interpreting the results. Because many of the studies used a multiple baseline across participants design with three participants, the presence or absence of an effect for each participant could have a large impact on the overall judgment of a functional relation. In addition, the magnitude of effect sizes cannot be compared directly between the RCTs and SCRD studies due to methodological differences in study types.

Moderator analyses revealed that effect sizes using context-bound outcome measures had a larger mean effect size than those with potentially context-bound or generalized outcome measures for the RCTs. This finding is consistent with those reported by Yoder et al. (2013) and Fuller and Kaiser (2020) for social communication outcomes in children with ASD. In addition, RCTs at risk for CME exhibited a significant, positive effect size, but those free from CME risk did not. These results for the role of boundness and CME risk could not be replicated for the SCRD studies because all of the SCRD study effect sizes were context-bound and at risk for CME. Although some of the SCRD studies did include generalization probes (e.g., with a different communication partner or setting), in the vast majority of cases probes were not frequent enough to meet quality standards for inclusion. Similarly, very few effect sizes from the SCRD studies included distal outcome measures. Thus, proximity of the outcome measure could not be tested for the SCRD studies. Results for the RCTs and SCRD were consistent for publication bias being identified by the Egger’s test but not the moderator analysis. The relatively small number of unpublished studies for both types of studies limited the moderator analysis. For both RCTs and SCRD studies, we were unable to test for a moderating effect of time in intervention, despite attempts to use multiple intensity variables. Too few studies included key details about intensity in the included reports.

In sum, these findings provide support for the use of responsivity intervention strategies for children with ASD for improving prelinguistic and language skills. As expected, findings are more robust for context-bound outcome measures than other types of outcome measures (e.g., potentially context-bound and generalized characteristics).

### Limitations

Limitations for meta-analyses are influenced by primary study level characteristics as well as meta-analytic level characteristics. For the current study, imprecise reporting at the primary study level, especially for the study’s intensity, limited analyses. Despite calls for improved reporting of intervention details, only about half of the RCTs and SCRD studies provided sufficient information to determine the time in intervention, a less precise variable than the cumulative intervention intensity (Warren et al., 2007). Given the potential importance of treatment intensity for the effectiveness, cost (financial and time), and feasibility of services for children, reports of future studies would be strengthened by explicit descriptions of intensity variables. Concerns of study quality of the included studies also influenced the meta-analysis. Forty-four SCRD design studies that would have otherwise been included were excluded because they failed to meet What Works Clearinghouse standards. Risk for CME was very common across included studies and should be attended to in future studies to minimize risk for bias.

The use of meta-analytic analyses for SCRD studies is a relatively new and still developing area. As a result, only studies that used a multiple baseline across participants design were able to be included in the current quantitative meta-analysis. Future meta-analyses on responsivity intervention strategies should be considered as other analytic approaches develop. Other limitations at the meta-analytic level include the potential failure to include relevant effect sizes and only including studies written in English. The risk of missing relevant effect sizes was minimized through multiple supplementary searches and completion of reliability checks at all screening levels. Lastly, robust variance estimation is most effective with at least 40 studies. Our analyses using robust variance estimation included 33 RCTs and 34 SCRD studies.

### Strengths

Although our searches yielded effect sizes from fewer than 40 RCTs or SCRD studies, the use of robust variance estimation remains a strength of this meta-analysis. Robust variance estimation permits the inclusion of multiple effect sizes per study, which eliminates the loss of potentially important effect sizes. Second, we include both RCTs and SCRD studies, which is currently rare for systematic reviews and meta-analyses. This approach provides a more comprehensive review of the current literature base and opportunities for replication across the two study types. Third, we enhanced the quality of this meta-analysis by conducting interrater reliability for all screening levels and having two independent coders extract data (including risk of bias) for all included reports. Fourth, we considered the quality of the included studies through multiple avenues. We not only required studies to meet certain characteristics to be included, but also coded for study quality features including risk for bias and CME.

### Clinical Implications

This systematic review and meta-analysis provides empirical support for the use of responsivity intervention strategies to improve prelinguistic and language skills of children with ASD. Because the data are more robust for context-bound outcome measures (e.g., behaviors that occur during the intervention or a very similar setting) than generalized characteristics (e.g., use of targeted skills in a novel setting with someone other than the interventionist), gains in generalized characteristics should be monitored closely during clinical practice. The observed benefits of responsivity intervention strategies were observed for a wide variety of outcome measures (e.g., joint attention, use of gestures, verbal utterances, and vocalizations), which suggests that these strategies have broad application including both prelinguistic and early language skills.

### Research Implications

Additional, high-quality intervention studies regarding the observed benefits of responsivity intervention strategies are needed to further delineate the specific impact of these strategies and how features of such interventions can be adjusted to maximize gains. At the primary study level, future studies would be enhanced by continued improvement of study quality, especially minimizing risk for bias and CME, and more explicit reporting of putative moderators of treatment effects.

The need to report intensity data was especially apparent. Not only is such data needed to determine whether more intensive intervention is likely to have positive or negative effects on child outcomes, but also to control for intensity when investigating the role of other putative moderators, such as interventionist. Explicit reporting will improve the effectiveness of future meta-analytic moderator analyses. Primary studies that directly address the effect of intensity on intervention are also needed.

Continued inclusion of distal outcome measures in coordination with proximal measures is also warranted. Explicitly identifying outcomes as proximal versus distal will allow readers to accurately weigh the results. Because distal measures are expected to yield smaller effect sizes than proximal measures, achieving a relatively large effect size for a distal measure should be noted. SCRD studies can be used within a programmatic line of research to guide selection of outcome measures in RCTs. For example, an SCRD may include some generalization and maintenance data with sufficient data points to determine whether those dependent variables may be suitable distal and/or generalized characteristic outcome measures for a subsequent RCT. The evidence base would also benefit from the inclusion of studies that provide specific responsivity intervention strategies outside of large treatment packages as well as explicit descriptions of strategies implemented. Such evidence would facilitate ongoing efforts to identify active ingredients of interventions and inform modifications aimed at increasing effectiveness.

## Conclusions

This meta-analysis provides support for the use of responsivity intervention strategies with young children with ASD to support growth in prelinguistic and language skills. Positive results were observed for both RCTs and SCRD studies. Moderator analysis indicated the need to attend to the potential roles of CME and boundedness of outcome measures. Concerns of study quality, risk for bias, and omission of key intervention details were also observed. These findings can be applied to future studies to enhance the quality of the literature base and the confidence of clinical recommendations for intervention practices.

## Supplementary Information

Below is the link to the electronic supplementary material.

Supplementary file1 (PDF 9 kb)

Supplementary file2 (PDF 99 kb)
