# Supplementary material for: Effectiveness of Responsivity Intervention Strategies on Prelinguistic and Language Outcomes for Children with Autism Spectrum Disorder: A Systematic Review and Meta-Analysis of Group and Single Case Studies
Source: J Autism Dev Disord. 2021 Nov 15;52(11):4783–816. doi: 10.1007/s10803-021-05331-y (PMC9556387; doi:10.1007/s10803-021-05331-y)
Supplement: Supplementary file 1 — Supplementary file1 (PDF 9 kb) [file 10803_2021_5331_MOESM1_ESM.pdf]

Supplementary Information 1

Search Strategy for ProQuest Databases

MJMAINSUBJECT.EXACT("Autism Spectrum Disorders") AND ti,ab,su(infants OR infant OR children OR preschool OR preschoolers OR toddlers) AND ti,ab,su(responsiveness OR responsivity OR responsive OR contingent OR contingency OR contingently OR recast* OR "linguistic mapping" OR interaction OR communication OR "early start denver model" or "joint attention" OR JASPER or imitate or imitation) AND ti,ab,su(intervention OR treatment OR therapy OR treat* OR teach OR teaching) AND (language OR vocabulary OR speech OR expressive OR comprehension OR spoken OR verbalization OR verbalizations OR vocalization OR vocalizations OR communicate OR communication)
