# Supplementary material for: Effectiveness of Responsivity Intervention Strategies on Prelinguistic and Language Outcomes for Children with Autism Spectrum Disorder: A Systematic Review and Meta-Analysis of Group and Single Case Studies
Source: J Autism Dev Disord. 2021 Nov 15;52(11):4783–816. doi: 10.1007/s10803-021-05331-y (PMC9556387; doi:10.1007/s10803-021-05331-y)
Supplement: Supplementary file 2 — Supplementary file2 (PDF 99 kb) [file 10803_2021_5331_MOESM2_ESM.pdf]

Supplementary Information 2

Single Case Research Design Studies Excluded Due to Failure to Meet Quality Standards

Akamoglu, Y. (2019). *Storybook reading with young children with autism: A parent-implemented communication intervention.* (Publication No., AAI13804642). [Doctoral dissertation, University of Illinois at Urbana-Champaign]. ProQuest.

Bissinger, E. A. (2010). *Social intervention and play for children with autism spectrum disorders.* (Publication No., AAI3368465). [Doctoral dissertation, Northeastern University]. ProQuest.

Bradshaw, J., Koegel, L. K., & Koegel, R. L. (2017). Improving functional language and social motivation with a parent-mediated intervention for toddlers with autism spectrum disorder. *Journal of Autism and Developmental Disorders, 47*(8), 2443-2458. https://doi.org/10.1007/s10803-017-3155-8

Brain, T., & Pat, M. (2019). Effectiveness of a low-intensity peer-mediated intervention for middle school students with autism spectrum disorder. *Research in Autism Spectrum Disorders, 62*, 26-38. https://doi.org/10.1016/j.rasd.2019.02.003

Brown, J. A., & Woods, J. J. (2015). Effects of a triadic parent-implemented home-based communication intervention for toddlers. *Journal of Early Intervention, 37*(1), 44-68. https://doi.org/10.1177/1053815115589350

Bruinsma, Y. E. M. (2005). *Increases in the joint attention behavior of eye gaze alternation to share enjoyment as a collateral effect of Pivotal Response Treatment for three children with autism.* (Publication No. AAI3145711). [Doctoral dissertation, University of California, Santa Barbara]. ProQuest.

Cafiero, J. M. (1996). *Teaching parents of children with autism Picture Communication Symbols as a natural language to decrease levels of family stress.* (Publication No. AAM9540360). [Doctoral dissertation, University of Toledo]. ProQuest.

Charlop-Christy, M. H., & Carpenter, M. H. (2000). Modified incidental teaching sessions: A procedure for parents to increase spontaneous speech in their children with autism. *Journal of Positive Behavior Interventions, 2*(2), 98-112. https://doi.org/10.1177/109830070000200203

Franco, J. H. (2010). *Teaching prelinguistic communication skills to school age children with autism.* (Publication No. AAI3391111). [Doctoral dissertation, University of Texas at Austin]. ProQuest.

Franco, J. H., Davis, B. L. & Davis, J. L. (2013). Increasing social interaction using prelinguistic milieu teaching with nonverbal school-age children with autism. *American Journal of Speech Language Pathology, 22*(3), 489-502. https://doi.org/10.1044/1058-0360(2012/10-0103)

Hancock, T. B., & Kaiser, A. P. (2002). The effects of trainer-implemented enhanced milieu teaching on the social communication of children with autism. *Topics in Early Childhood Special Education, 22*(1), 39-54. https://doi.org/10.1177/027112140202200104

Ingersoll, B., & Gergans, S. (2007). The effect of a parent-implemented imitation intervention on spontaneous imitation skills in young children with autism. *Research in Developmental Disabilities, 28*(2), 163-175. https://doi.org/10.1016/j.ridd.2006.02.004

Ingersoll, B., Meyer, K., Bonter, N., & Jelinek, S. (2012). A comparison of developmental social-pragmatic and naturalistic behavioral interventions on language use and social engagement in children with autism. *Journal of Speech Language Hearing Research, 55*(5), 1301-1313. https://doi.org/10.1044/1092-4388(2012/10-0345)

Ingersoll, B. R., Wainer., A. L., Berger, N. I., & Walton, K. M. (2017). Efficacy of low intensity, therapist-implemented Project ImPACT for increasing social communication skills in young children with ASD. *Developmental Neurorehabilitation, 20*(8), 502-510. https://doi.org/10.1080/17518423.2016.1278054

Kaiser, A. P., Hancock, T. B., & Nietfeld, J. P. (2000). The effects of parent-implemented Enhanced Milieu Teaching on the social communication of children who have autism. *Early Education and Development, 11*(4), 423-446. https://doi.org/10.1207/s15566935eed1104_4

Kashinath, S., Woods, J., & Goldstein, H. (2006). Enhancing generalized teaching strategy use in daily routines by parents of children with autism. *Journal of Speech, Language, & Hearing Research, 49*(3), 466-485. https://doi.org/10.1044/1092-4388(2006/036)

Kinard, J. L. (2016). *Start that coexistence: Three studies of parent responsiveness to children with or at-risk for autism spectrum disorder.* (Publication No. AAI3703825). [Doctoral dissertation, The University of North Carolina at Chapel Hill]. ProQuest.

Knap, K. A. (2018). *Parent-child interaction therapy for children with autism spectrum disorder: An analysis of behavioral patterns and treatment barriers.* (Publication No., AAI10838657). [Doctoral dissertation, University of South Florida]. ProQuest.

Koegel, R., Bradshaw, J., Ashbaugh, K., & Koegel, L. K. (2014). Improving question-asking initiations in young children with autism using pivotal response treatment. *Journal of Autism and Developmental Disorders, 44*(4), 816-827. https://doi.org/10.1007/s10803-013-1932-6

Koegel, R. L., O'Dell, M. C., & Koegel, L. K. (1987). A natural language teaching paradigm for nonverbal autistic children. *Journal of Autism and Developmental Disorders, 17*(2), 187-200. https://doi.org/10.1007/BF01495055

Koegel, R. L., Symon, J. B., & Koegel, L. K. (2002). Parent education for families of children with autism living in geographically distant areas. *Journal of Positive Behavior Interventions, 4*(2), 88-103. https://doi.org/10.1177/109830070200400204

Koegel, R. L., Vernon, T. W., & Koegel, L. K. (2009). Improving social initiations in young children with autism using reinforcers with embedded social interactions. *Journal of*

Autism & Developmental Disorders, 39(9), 1240-1251. https://doi.org/10.1007/s10803-009-0732-5

Kroeger, K. A., & Nelson, W. M., III. (2006). A language programme to increase the verbal production of a child dually diagnosed with Down syndrome and autism. *Journal of Intellectual Disability Research, 50*(2), 101-108. https://doi.org/10.1111/j.1365-2788.2005.00734.x

Matsuzaki, A., & Yamamoto, J. (2012). Effects of an early intervention program on preverbal communication in a child with autism: Developmental and behavioral analysis with a multiple-baseline design. *Japanese Journal of Special Education, 49*(6), 657-669. https://doi.org/10.6033/tokkyou.49.657

McNerney, E. K. (2003). *Videotape communication between school and clinic and the effects on teacher behavior and generalization of expressive language to classroom settings for children with autism.* (Publication No. AAI3093300). [Doctoral dissertation, University of California, Santa Barbara]. ProQuest.

Meadan, H., Snodgrass, M. R., Meyer, L. E., Fisher, K. W., Chung, M. Y., & Halle, J. W. (2016). Internet-based parent-implemented intervention for young children with autism: A pilot study. *Journal of Early Intervention, 38*(1), 3-23. https://doi.org/10.1177/1053815116630327

Mrachko, A. A. (2016). *Teaching paraprofessionals to implement a social communication intervention for young children with ASD.* (Publication No. AAI3735330). [Doctoral dissertation, University of Pittsburgh]. ProQuest.

Nunes, D. R. P., Araujo, E. R., Walter, E., Soares, R., & Mendonça, C. (2016). Augmenting caregiver responsiveness: An intervention proposal for youngsters with autism in Brazil. *Early Childhood Education Journal, 44*(1), 39-49. https://doi.org/10.1007/s10643-014-0677-y

Olive, M. L., de la Cruz, B., Davis, T. N., Chan, J. M., Lang, R. B., O'Reilly M. F., & Dickson, S. M. (2007). The effects of enhanced milieu teaching and a voice output communication aid on the requesting of three children with autism. *Journal of Autism and Developmental Disorders, 37*(8), 1505-1513. https://doi.org/10.1007/s10803-006-0243-6

Oliver, K. A. E. (2019). *Following parent lead: Outcomes of a brief, individualized pivotal response treatment education program for parents of children newly diagnosed with autism.* (Publication No. AAI10828005). [Doctoral dissertation, University of California, Santa Barbara]. ProQuest.

Pierce, K., & Schreibman, L. (1995). Increasing complex social behaviors in children with autism: effects of peer-implemented pivotal response training. *Journal of Applied Behavior Analysis, 28*(3), 285-295. https://doi.org/10.1901/jaba.1995.28-285

Ragusa, A. H. (2018). *Effects of classroom Milieu Communication Therapy on prelinguistic and play skills of preschoolers with autism spectrum disorder.* (Publication No. 10285454) [Doctoral dissertation, Hofstra University]. ProQuest.

Robinson, S. E. (2011). Teaching paraprofessionals of students with autism to implement pivotal response treatment in inclusive school settings using a brief video feedback training package. *Focus on Autism and Other Developmental Disabilities, 26*(2), 105-118. https://doi.org/10.1177/1088357611407063

Rocha, M. L. (2012). *Teaching child-initiated social interactions to preverbal children with autism: Effects on social initiations, treatment response profiles and vocal communication.* (Publication No. 3454525). [Doctoral dissertation, University of California, San Diego]. ProQuest.

Rogers, S. J., Hayden, D., Hepburn, S., Charlifue-Smith, R., Hall, T., & Hayes, A. (2006). Teaching young nonverbal children with autism useful speech: a pilot study of the Denver Model and PROMPT interventions. *Journal of Autism and Developmental Disorders, 36*(8), 1007-1024. https://doi.org/10.1007/s10803-006-0142-x

Schepis, M. M., Reid, D. H., Behrmann, M. M., & Sutton, K. A. (1998). Increasing communicative interactions of young children with autism using a voice output communication aid and naturalistic teaching. *Journal of Applied Behavior Analysis, 31*(4), 561-578. https://doi.org/10.1901/jaba.1998.31-561

Smith, A. E., & Camarata, S. (1999). Using teacher-implemented instruction to increase language intelligibility of children with autism. *Journal of Positive Behavior Interventions, 1*(3), 141-151. https://doi.org/10.1177/109830079900100302

Symon, J. B. (2005). Expanding interventions for children with autism: Parents as trainers. *Journal of Positive Behavior Interventions, 7*(3), 159-173. https://doi.org/10.1177/10983007050070030501

Trembath, D., Balandin, S., Togher, L., & Stancliffe, R. J. (2009). Peer-mediated teaching and augmentative and alternative communication for preschool-aged children with autism. *Journal of Intellectual Developmental Disabilities, 34*(2), 173-186. https://doi.org/10.1080/13668250902845210

Verschuur, R., Huskens, B., Verhoeven, L., & Didden, R. (2017). Increasing opportunities for question-asking in school-aged children with autism spectrum disorder: Effectiveness of staff training in pivotal response treatment. *Journal of Autism and Developmental Disorders, 47*(2), 490-505. https://doi.org/10.1007/s10803-016-2966-3

Vismara, L. A., Colombi, C., & Rogers, S. J. (2009). Can one hour per week of therapy lead to lasting changes in young children with autism? *Autism: The International Journal of Research & Practice, 13*(1), 93-115. https://doi.org/10.1177/1362361307098516

Vismara, L. A., McCormick, C., Young, G. S., Nadhan, A., & Monlux, K. (2013). Preliminary findings of a telehealth approach to parent training in autism. *Journal of Autism and Developmental Disorders, 43*(12), 2953-2969. https://doi.org/10.1007/s10803-013-1841-8

Zaghlawan, H. (2012). *A parent-implemented intervention to improve spontaneous imitation by young children with autism.* (Publication No. 3496689) [Doctoral dissertation, University of Illinois at Urbana-Champaign]. ProQuest.

Zaghlawan, H. Y., & Ostrosky, M. M. (2016). A parent-implemented intervention to improve imitation skills by children with autism: A pilot study. *Early Childhood Education Journal, 44*(6), 671-680. https://doi.org/10.1007/s10643-015-0753-y
